# Understanding the Liquid Structure in Mixtures of Ionic Liquids with Semiperfluoroalkyl or Alkyl Chains

**DOI:** 10.1021/acs.jpcb.3c02647

**Published:** 2023-08-09

**Authors:** Naomi S. Elstone, Karina Shimizu, Emily V. Shaw, Paul D. Lane, Lucía D’Andrea, Bruno Demé, Najet Mahmoudi, Sarah E. Rogers, Sarah Youngs, Matthew L. Costen, Kenneth G. McKendrick, Jose N. Canongia Lopes, Duncan W. Bruce, John M. Slattery

**Affiliations:** † Department of Chemistry, University of York, Heslington, York YO10 5DD, U.K.; ‡ Centro de Química Estrutural, Institute of Molecular Sciences, Instituto Superior Técnico, Universidade de Lisboa, Av. Rovisco Pais, Lisboa 1049 001, Portugal; § Institute of Chemical Sciences, School of Engineering and Physical Sciences, Heriot−Watt University, Edinburgh EH14 4AS, U.K.; ∥ Institut Laue−Langevin, Grenoble 38000, France; ⊥ ISIS Neutron Source Facility, Harwell Science and Innovation Campus, Didcot OX11 0DE, U.K.

## Abstract

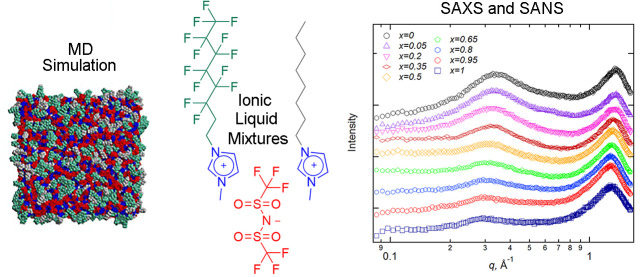

By mixing ionic liquids (ILs), it is possible to fine-tune their bulk and interfacial structure. This alters their physical properties and solvation behavior and is a simple way to prepare a collection of ILs whose properties can be tuned to optimize a specific application. In this study, mixtures of perfluorinated and alkylated ILs have been prepared, and links between composition, properties, and nanostructure have been investigated. These different classes of ILs vary substantially in the flexibility and polarizability of their chains. Thus, a range of useful structural and physical property variations are accessible through mixing that will expand the library of IL mixtures available in an area that to this point has received relatively little attention. In the experiments presented herein, the physical properties and bulk structure of mixtures of 1-methyl-3-octylimidazolium bis(trifluoromethylsulfonyl)imide [C_8_MIM][Tf_2_N] and 1-(1*H*,1*H*,2*H*,2*H*-perfluorooctyl)-3-methylimidazolium bis(trifluoromethylsulfonyl)imide [C_8_MIM-F_13_][Tf_2_N] have been prepared. The bulk liquid structure was investigated using a combination of small-angle X-ray and neutron scattering (SAXS and SANS, respectively) experiments in combination with atomistic molecular dynamics simulations and the measurement of density and viscosity. We observed that the addition of [C_8_MIM-F_13_][Tf_2_N] to [C_8_MIM][Tf_2_N] causes changes in the nanostructure of the IL mixtures that are dependent on composition so that variation in the characteristic short-range correlations is observed as a function of composition. Thus, while the length scales associated with the apolar regions (polar non-polar peak—PNPP) increase with the proportion of [C_8_MIM-F_13_][Tf_2_N] in the mixtures, perhaps surprisingly given the greater volume of the fluorocarbon chains, the length scale of the charge-ordering peak decreases. Interestingly, consideration of the contact peak shows that its origins are both in the direct anion···cation contact length scale and the nature (and hence volume) of the chains appended to the imidazolium cation.

## Introduction

Ionic liquids (ILs) have garnered a great amount of interest in recent years in a wide range of applications.^1^ They have particular relevance as reaction media for catalysis and have been used at the pilot scale as supported IL phases for a range of gas-phase reactions and at the industrial scale for hydrogenation reactions using a solid catalyst with an IL layer.^2,3^ One of the advantages of ILs over molecular solvents is their tunability, as one or both of the cations or anions can be varied, leading to a very wide range of potential solvent properties. Yet, it is exactly the huge number of potential materials that also poses a challenge, as making and testing vast numbers of individual ILs to find an ideal liquid for the desired application is prohibitive in both time and cost.^4^ This issue is addressed effectively by preparing IL mixtures,^5^ where properties can be tuned smoothly in liquids created from a much smaller number of starting ILs. A small library of pure ILs can be made quickly and cheaply and then used to generate a large range of mixtures that cover a spectrum of physical properties, interfacial and bulk liquid structures.^6−8^ The ability to control physical properties such as viscosity, conductivity, and polarity allows ILs to be optimized for applications including electrolytes in batteries,^9,10^ carbon capture,^11,12^ biomass processing,^13,14^ and catalysis.^15−17^ Fluoroalkyl-functionalized ILs have properties which are distinct from the more commonly explored alkyl-functionalized ILs. For this reason, they have been explored for applications in the recovery and recycling of perfluorinated contaminants,^18−20^ gas storage,^21,22^ gas separation,^23^ and materials preparation including nanoreactors^24^ and solar cells.^25^ Recent studies have shown that physical properties and bulk structure are often correlated.^26^ Understanding of bulk structure has potential for templating applications and optimization of catalysis.

While still a relatively underexplored area of ILs when compared to the pure materials, in recent years, IL mixtures have generated substantial interest. Studies have been carried out where the anion is varied, while the cation remains constant^27,28^ and also where the anion remains constant, while the structure of the cation changes (most commonly achieved *via* variation in its chain length).^29−31^ In the present study, the anion is kept constant, and the *nature* of the alkyl chain appended to the cation, rather than its length, is varied to give mixtures with differing behavior. This represents a rarely explored approach.

**Figure 1:**
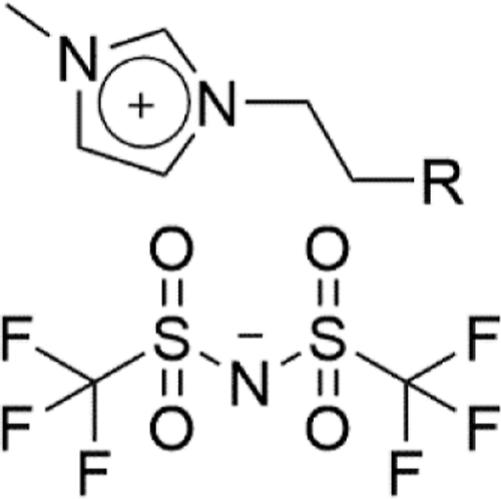
General structure of the materials under investigation in this work. R = H, C_*n*_H_2*n*+1_, or C_*n*_F_2*n*+1_ as defined in the text.

We have previously studied the bulk and interfacial structure of mixed IL systems prepared from 1-methyl-3-alkyl-imidazolium ILs ([C_*n*_MIM][Tf_2_N]) with various alkyl chain lengths.^6,7^ Investigations of the bulk structure of mixtures of [C_12_MIM][Tf_2_N] (Figure 1—R = C_10_H_21_) in [C_2_MIM][Tf_2_N] (Figure 1—R = H) using small-angle neutron scattering (SANS) and small-angle X-ray scattering (SAXS) showed that at low concentrations of the long-chain component, small aggregates of the cation dodecyl chains were observed. Further, as the composition moved toward pure [C_12_MIM][Tf_2_N], a percolated nanostructure formed, where the polar parts of the liquid comprising the anion and imidazolium headgroup phase separated from the dodecyl chains to form two distinct sub-phases. Complementary measurements at the vacuum–liquid interface in the same mixtures using reactive atom scattering, combined with laser-induced fluorescence (RAS-LIF),^32^ found that [C_12_MIM][Tf_2_N] was preferentially enriched at the liquid surface.

Investigation of the bulk liquid structure of other mixtures [C_*n*_MIM]_1–*x*_[C_*m*_MIM]_*x*_[Tf_2_N] (*n*, *m* = 2, 4, 6, 8, and 10) showed similar trends where one component forms a percolated structure, while the other does not.^6^ Where both components have a percolated structure, the alkyl-rich region varies in size between the values recorded for the two pure components depending on the composition of the mixture. Indeed, a detailed SAXS study of mixtures of [C_8_MIM][Tf_2_N] and [C_12_MIM][Tf_2_N] reported that as the concentration of [C_12_MIM][Tf_2_N] increased, there was a non-linear increase in the size of the non-polar domain, with a positive deviation that could be fitted to a second-order polynomial. This was interpreted as the structure of [C_12_MIM][Tf_2_N] dominating the mixture, causing the non-polar region to be larger than expected if the structure varied in a linear manner with composition.^33^

We have also reported investigations into the interfacial composition and structure of mixtures formed between 1-methyl-3-octylimidazolium bis(trifluoromethylsulfonyl)imide (Figure 1—R = C_6_H_13_—[C_8_MIM][Tf_2_N]) and its semiperfluorinated analogue (Figure 1—R = C_6_F_13_—[C_8_MIM-F_13_][Tf_2_N]) where the chain in the 3-position is 1*H*,1*H*,2*H*,2*H*-perfluorooctyl (−CH_2_CH_2_C_6_F_13_).^8^ The use of the semiperfluorinated component is an alternative strategy to exert influence on both the nature of the gas–liquid interface and the structure of the bulk liquid. Such materials have the potential to expand the scope for the application of IL mixtures and could lead to advantageous gains outweighing the cost differential in starting materials. For example, Lepre *et al.* have reported on the differences in the solubility of perfluorocarbon gases in the two ILs just mentioned.^34^ Despite the tendency of hydrocarbon and perfluorocarbon chains to demix,^35,36^ the two components were miscible over the entire compositional range, and both RAS-LIF and surface tension data showed that the fluorinated cation is preferentially enriched at the surfaces of these mixtures.^8^

An investigation into the bulk structure of a related system ([C_8_MIM-F_13_]_*x*_[C_8_MIM]_1–*x*_Br) has been carried out using molecular dynamics (MD) simulations.^37,38^ This work proposed that, from the analysis of simulated scattering patterns, a triphilic structure forms when the perfluorinated and alkylated ILs are mixed at certain compositions. In the proposed structures, the polar domains formed from the ionic parts of the ILs, and the aliphatic domains formed from the hydrocarbon chains (as can be seen in many IL systems^39−43^) were augmented by an additional domain rich in fluorinated chains.^18^

Given the perhaps unexpected miscibility of [C_8_MIM][Tf_2_N] and [C_8_MIM-F_13_][Tf_2_N] found in our previous studies and the potential for the introduction of triphilic structures, it was therefore of interest to investigate this system in detail here. Volumetric (density) and transport properties (viscosity) were measured, in addition to exploring the nanostructure of the mixtures using SAXS and SANS measurements complemented by atomistic MD simulations.

## Experimental Section

All ILs investigated were synthesized in-house utilizing the methodologies reported in the Supporting Information, which were developed from those previously reported.^27^ Perdeuterated *d*_17_-1-bromooctane was provided by the ISIS deuteration laboratory and used without further purification.

### Scattering Measurements

SANS measurements were carried out on beamline D16 at the Institute Laue–Langevin, ILL, and SANS2D on Target Station 2 at the ISIS Pulsed Neutron and Muon Source. In order to get the best contrast between the ionic and non-polar regions of the ILs in the neutron experiments, mixtures were prepared using [C_8_MIM-F_13_][Tf_2_N] with either [C_8_MIM-*d*_17_][Tf_2_N] or [C_8_MIM][Tf_2_N], providing two contrasts for each composition. The samples were measured in 1 mm path length and 1 cm wide quartz Hellma cells.

#### Measurements Carried Out on D16

In order to achieve the desired *q*-range on D16, measurements were carried out at four angles to give a *q*-range of 0.08–1.6 Å^–1^. The data were processed using a combination of the ILL in-house software LAMP^44^ and the NCNR package for IGORPro.^45^ Further details of the beamline setup used can be found in the Supporting Information along with fitting parameters (Tables S4 and S5). Raw data are found at 10.5291/ILL-DATA.9-10-1720.

#### Measurements Carried Out on SANS2D

Measurements were carried out on SANS2D covering a *q*-range of 0.004–0.97 Å^–1^ and used a temperature-controlled sample changer set at 20 °C. Further details of the beamline setup used can be found in the Supporting Information. Raw data are found at 10.5286/ISIS.E.RB2220026-1.

#### SAXS Measurements

SAXS employed a Bruker D8 Discover diffractometer equipped with a bespoke temperature-controlled, bored-graphite rod furnace custom built at the University of York. Cu K_α_ (λ = 0.154056 nm) radiation was used, generated from a 1 mS microfocus source. Diffraction patterns were recorded on a 2048 × 2048 pixel Bruker VANTEC 500 area detector set at a distance of 121 mm from the sample, allowing simultaneous collection of small- and wide-angle scattering data. Mixtures used employed only hydrogenous components.

Fitting of the data was carried out using SASView,^46^ employing several Lorentzian peaks to describe the observed scattering peaks, with multiple iterations leading to the best fit to the data (Tables S6 and S7). In some cases, an Ornstein–Zernike model (described as a Lorentzian within the software) is also applied, when the background is not flat. The standard combination of functions used to achieve a good fit to all data collected is a combination of three “peak Lorentz” functions and a Lorentzian.

### MD Simulations

MD simulations were carried out using DL_POLY 2.20 and GROMACS 5.1.4 packages.^47−52^ The [C_8_MIM]_1–*x*_[C_8_MIM-F_13_]_*x*_[Tf_2_N] mixtures were modeled using the OPLS/AMBER-like CL&P force field.^26,27^ A scaling factor of α = 0.8 was applied to the partial charges of the charged part of the cation and the anion. The simulations started from low-density configurations with 2000 ion pairs built with fftool and Packmol software.^53,54^ The runs were performed using 2 fs timesteps and 1.2 nm cut-off distances, followed by a 10 ns simulated annealing scheme. In the annealing process, the temperature ranges from 300 to 550 K, V-rescale thermostat and Berendsen barostat relaxation times of 0.5 and 4 ps, respectively, and then brought down to 300 K temperature and 1 atm pressure. These simulations were equilibrated under isobaric isothermal ensemble conditions (*p* = 0.1 MPa and *T* = 300 K, with V-rescale thermostat and Berendsen barostat relaxation time constants of 0.5 and 2 ps, respectively) using 2 fs timestep and 1.6 nm cut-off distance for 25 ns. The density of each system reached a constant and consistent value after 10 ns, indicating that equilibrium had been attained, and possible ergodicity problems had been overcome. Finally, a 10 ns production stage was performed using 1 fs timestep in isothermal–isobaric ensemble conditions *p* = 0.1 MPa and *T* = 300 K, with Nosé–Hoover thermostat and Parrinello–Rahman barostat relaxation times of 0.5 and 4 ps, respectively. The final volumes of the simulation boxes were larger than 10 × 10 × 10 nm^3^. Pair correlation functions, *g*_*ij*_(*r*), and total structure factor functions, *S*(*q*), were calculated according to the formulas and methodologies described previously.^55^

### Density Measurements

Density measurements used an Anton Paar DSA 5000 vibrating tube densitometer at 20 °C, calibrated according to the manufacturer’s protocol, and each data point was calculated from the average of multiple runs. The manufacturer’s specification of accuracy in the measurements is ±1 μg cm^–3^, and their specified temperature accuracy is given as ±1 mK.

### Viscosity Measurements

Viscosity was measured with a programmable Brookfield rotational rheometer RVDV-II + Pro (cone and plate version) with temperature control (temperature accuracy ±1 K) using a cryostat. A Brookfield silicone fluid (97 mPa s, 298 K) was used as a NIST-traceable viscosity standard. The viscosity data were collected between 298 and 328 K using a CP-40 cone. The sample volume used for each experiment was ∼0.5 mL. The viscosity accuracy was ±1.0% of the full-scale viscosity range (FSVR), which has been calculated using the equation FSVR [mPa s] = (TK × SMC × 10,000)/rpm (where TK is the DV-II + Pro torque constant and SMC is the spindle multiplier constant). All fluids measured showed Newtonian behavior at 293 K within the range of shear rates examined (7.50–712.50 s^–1^).

Water content was measured for the pure ILs using a C20S compact Karl Fischer coulometric titrator. Known quantities of the pure ILs were dissolved in known volumes of CH_2_Cl_2_, and water contents for the solutions were compared to that of the solvent used to prepare them to allow the water content of the ILs to be calculated. The error reported by the manufacturer is ±0.1 μg.

## Results

### Synthesis

The IL [C_8_MIM-F_13_][Tf_2_N] was prepared by reaction of 1-methylimidazole with 1*H*,1*H*,2*H*,2*H*-perfluorooctyl iodide in acetonitrile under reflux for an extended period, leading initially to a dark, amber oil which is then purified as described in the Supporting Information. There are, of course, several ways to prepare such materials, but in our experience, the methods adopted, while longer and a little more involved, avoid impurities^56^ that can arise from the use of activated carbon^57^ and/or alumina^58,59^ in the purification. The purity of these materials is evidenced by the combination of data from NMR spectroscopy, combustion analysis, Karl Fischer titration, and halide testing using AgNO_3_. The resulting materials do retain very small amounts of colored impurities, which must have extremely high molar absorption coefficients, as their presence does not adversely influence characterization. The judgement applied is that these impurities are preferable to those that may be introduced by decolorization protocols, especially for scattering studies where small, particulate impurities (*e.g.*, alumina or activated carbon) would affect the data significantly.

### Density

Mixtures with composition [C_8_MIM]_1–*x*_[C_8_MIM-F_13_]_*x*_[Tf_2_N] were prepared for *x* = 0.05, 0.2, 0.35, 0.5, 0.65, 0.8, and 0.95, and their densities were measured, from which molar volumes were calculated according to eq 1 in which *M*_*x*_ represents the weighted molar mass of the mixture for the particular value of *x*.
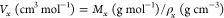
1

**Figure 2:**
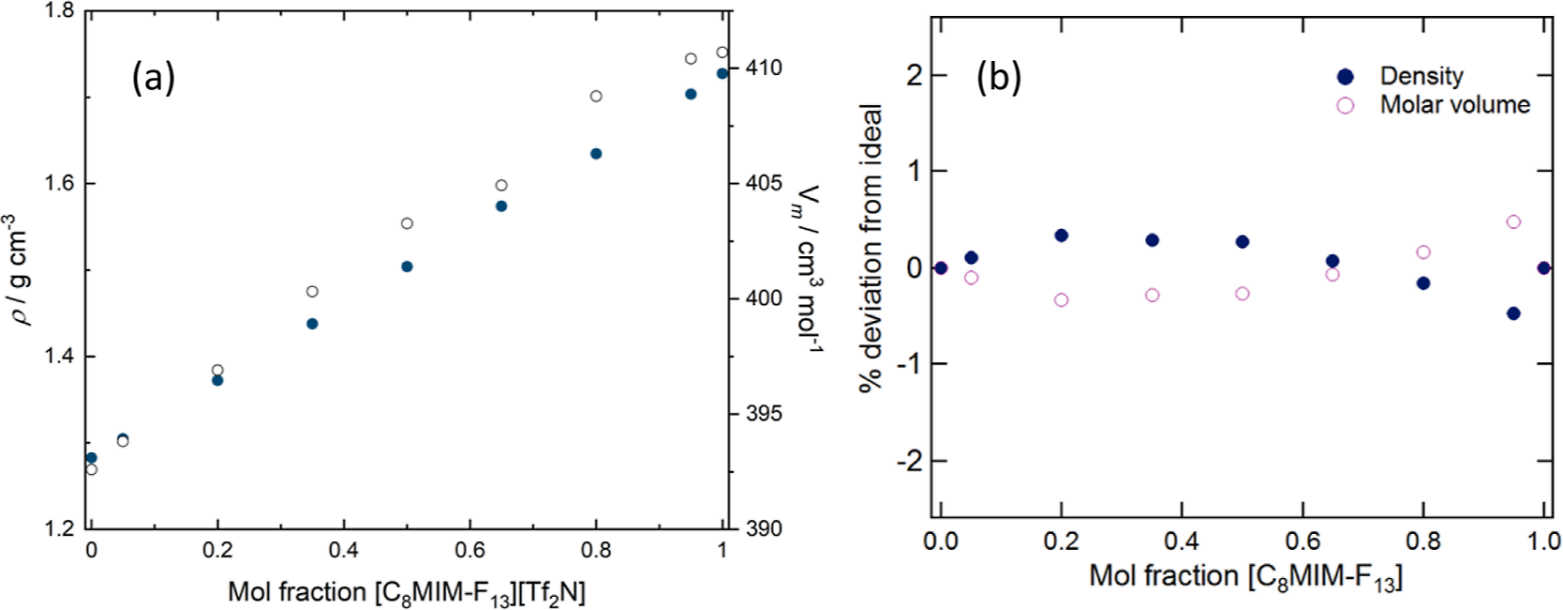
(a) Plot of density and molar volume as a function of composition and (b) deviation from linearity in density and molar volume for the mixtures [C_8_MIM]_1–*x*_[C_8_MIM-F_13_]_*x*_[Tf_2_N] at 20 °C; errors are small and within the markers.

The density data (Table S1 and plotted in Figure 2a) for each mixture were compared to the ideal densities/molar volumes calculated from the pure components using a linear mixing law. Percentage deviations are shown in Figure 2b and highlight that the deviation from ideality is small (<1%), which is likely to be similar to the experimental uncertainty (the main sources of error relating to sample preparation rather than instrument error). These small deviations from ideality are similar to data reported for other IL mixtures and indicate that there are no new strong interactions present in the liquid when the two components are mixed.^60^ The signs of the deviations suggest a slightly more efficient packing of the ions in the mixtures than would be expected based on their compositions, which is consistent with changes in the average inter-ion distances seen in the scattering and MD data (*vide infra*).

### Viscosity

**Figure 3:**
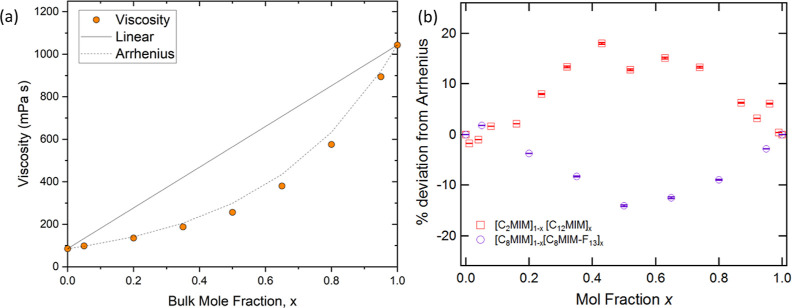
(a) Viscosity plotted against the composition of mixtures [C_8_MIM]_1–*x*_[C_8_MIM-F_13_]_*x*_[Tf_2_N]; the solid line indicates the variation in viscosity predicted by a linear mixing law and the dotted line that predicted using the Arrhenius model. Error bars are small and within the markers. (b) Percentage deviation of the measured viscosity from the Arrhenius model for the mixtures [C_8_MIM]_1–*x*_[C_8_MIM-F_13_]_*x*_[Tf_2_N] and [C_2_MIM]_1–*x*_[C_12_MIM]_*x*_[Tf_2_N].^32^

Figure 3 shows that the viscosity of [C_8_MIM-F_13_][Tf_2_N] at 298 K is approximately an order of magnitude higher than that of [C_8_MIM][Tf_2_N]. The viscosities of the mixtures were compared to both a linear mixing law and the Arrhenius mixing law (eq 2), which is often found to better describe the viscosity of binary liquid mixtures based on the viscosities of the pure components^61^
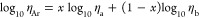
2where η_Ar_ is the calculated viscosity and η_a_ and η_b_ are the viscosities of the pure components of the system. Variable-temperature data are shown in Figures S1–S5 and show the expected reduction in viscosity with temperature.

The linear mixing law describes the system poorly, as expected, but interestingly, there is also a significant negative deviation from the Arrhenius mixing law, *i.e.*, [C_8_MIM]_1–*x*_[C_8_MIM-F_13_]_*x*_[Tf_2_N] mixtures are more fluid than would be expected based on the viscosities of the pure ILs (Figure 3a). This is the opposite trend to that observed when mixing imidazolium ILs with long (dodecyl) and short (ethyl) hydrocarbon chains,^32^ where a positive deviation was observed. Interestingly (Figure 3b), the absolute magnitude of the deviations in both examples is similar. The positive deviation was attributed to the development of a percolated nanostructure containing both polar and non-polar domains as the concentration of the long-chained IL increased, with the emergence of this self-organized structure providing some additional resistance to a shearing force. As described below and in contrast to the dodecyl/ethyl system, both pure ILs in the [C_8_MIM]_1–*x*_[C_8_MIM-F_13_]_*x*_[Tf_2_N] system have a percolated nanostructure, and so, the negative deviation from the Arrhenius mixing law presumably has a different origin, *e.g.*, it could imply that the percolated structure is disrupted on mixing. These observations, and thus the mixtures, are somewhat different from previous reports where there is good agreement with the Arrhenius model in IL mixtures, albeit those where neither component has a percolated structure as a pure material.^32,62^ Interestingly, the observed negative deviation means that mixtures with a significant proportion of the semiperfluorinated IL can be prepared that will have viscosities only marginally higher than that of the hydrogenous host IL. This is potentially very useful in applications where higher viscosity is a limiting factor.

### Scattering Experiments

**Figure 4:**
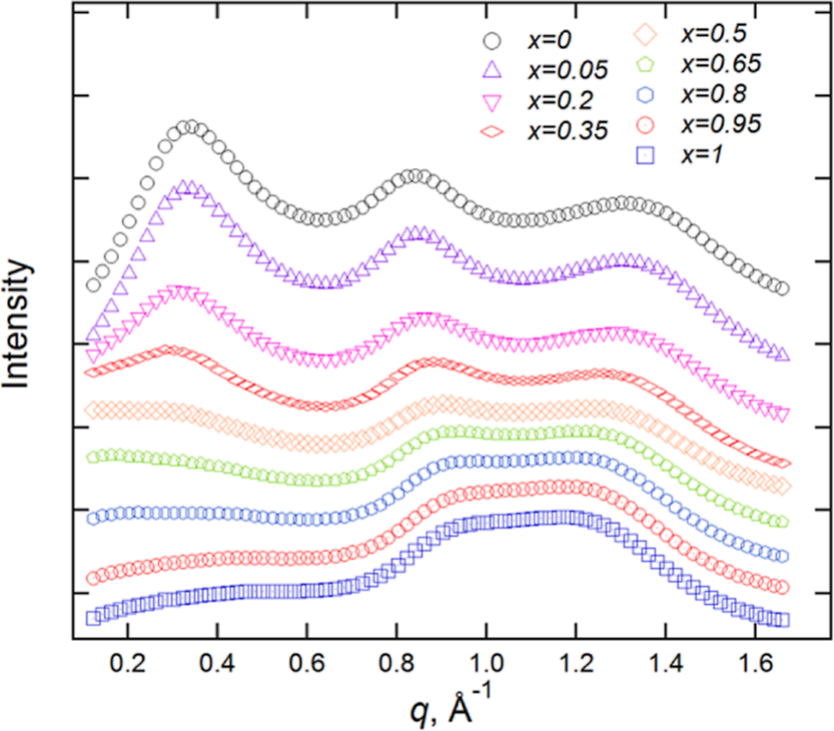
SAXS data for [C_8_MIM]_1–*x*_[C_8_MIM-F_13_]_*x*_[Tf_2_N] mixtures recorded at room temperature. Data have been offset in the *y*-axis to aid visualization.

**Figure 5:**
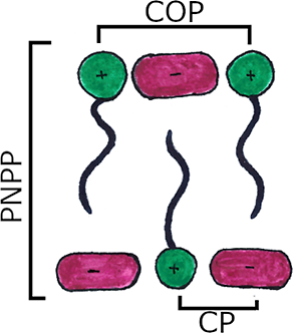
Cartoon illustration of the characteristic distances present in ILs.

Data from SAXS experiments on the [C_8_MIM]_1–*x*_[C_8_MIM-F_13_]_*x*_[Tf_2_N] mixtures at different compositions, shown in Figure 4, exhibit the three broad characteristic Bragg peaks that describe short-range ordering in ILs. These are the contact peak (CP) at *q* ≈ 1.3 Å^–1^ (*ca.* 4.8 Å) often regarded as representing direct anion–cation correlations and the charge-ordering peak (COP) at *q* ≈ 0.8 Å^–1^ (*ca.* 0.78 Å) commonly interpreted as corresponding to cation–anion–cation and anion–cation–anion correlations. In addition, at smaller values of *q* (longer length scales)—here <0.4 Å^–1^ (≥16 Å)—a polar non-polar peak (PNPP) can be seen. This is indicative of the presence of a bilayer structure with short-range order resulting from local separation of the polar (anion and cationic headgroup) and non-polar (chains) components (Figure 5) and accompanies the formation of a percolated structure.^6,7^

The SAXS pattern for pure [C_8_MIM][Tf_2_N] shows all three Bragg peaks clearly, whereas for pure [C_8_MIM-F_13_][Tf_2_N], the PNPP is absent. In the mixtures, the PNPP reduces in intensity as the proportion of [C_8_MIM-F_13_][Tf_2_N] in the mixtures increases. The separation between the CP and COP also reduces as the mixture becomes rich in the fluorinated material, leading to a significant overlap in their reflections as shown in Figure 4.

The decrease in the intensity of the PNPP in mixtures as the proportion of [C_8_MIM-F_13_][Tf_2_N] increases and the reason it is absent in [C_8_MIM-F_13_][Tf_2_N] itself can be attributed to X-ray contrast matching. Table 1 gives the calculated scattering length densities (SLDs) for the components of the ILs, which show that the SLDs for the ionic regions of the ILs and the perfluorinated tails are of similar magnitudes.

**Table 1:** Estimated SLDs for the Component Parts of the ILs of Interest, Calculated Using Available Bulk Densities^63^

	X-ray SLD (×10^–6^ Å^–2^)	neutron SLD (×10^–6^ Å^–2^)
[C_2_MIM][Tf_2_N]	13.2	2.41
[C_2_MIM]^+^	14.4	1.76
[Tf_2_N]^−^	12.6	2.79
C_8_H_17_	7.31	–0.41
C_8_D_17_	7.31	6.64
C_8_F_13_H_4_	13.7	3.20

**Figure 6:**
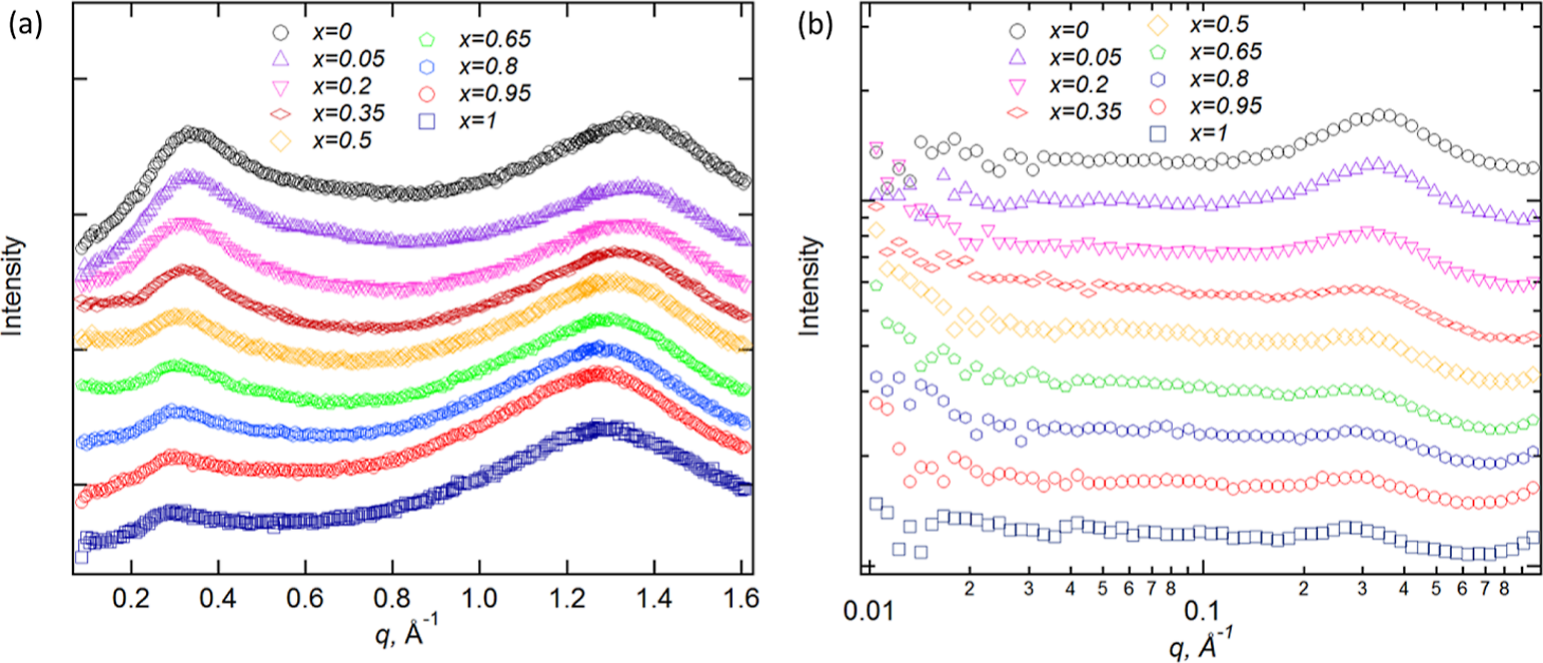
SANS data for [C_8_MIM-*d*_17_]_*x*_[C_8_MIM-F_13_]_1–*x*_[Tf_2_N] at room temperature, measured (a) on D16 and (b) on SANS2D (the logarithmic *x*-axis is employed here as the *q*-range spanned is larger and this allows features at low-*q* to be seen more clearly). Data have been offset in the *y*-axis to aid visualization, and alternative neutron contrasts are shown in Figures S5 and S6.

By contrast, SANS measurements for the pure liquids and their mixtures (Figure 6a,b) show a clear PNPP for all samples, confirming that contrast matching is the root cause of its absence in the SAXS data.

**Figure 7:**
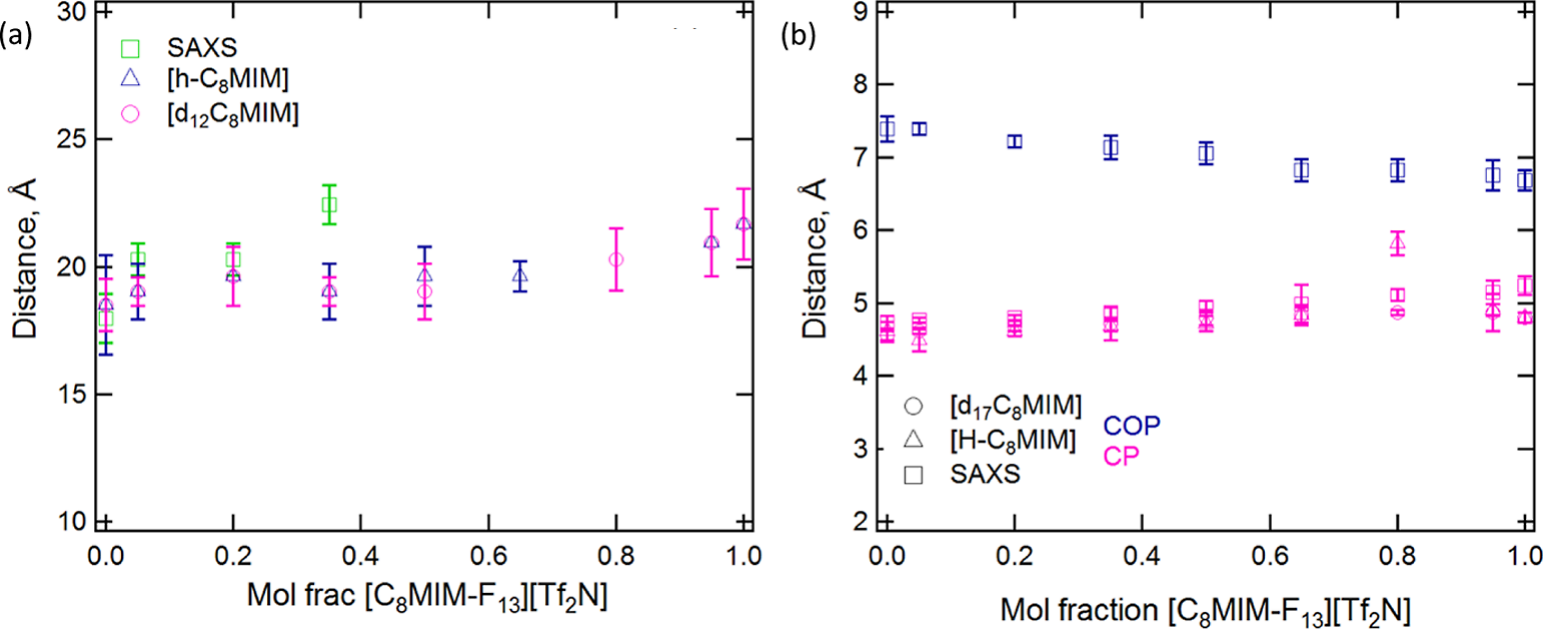
Variation in the three characteristic distances, *d*, present in the mixed IL structure as the composition of [C_8_MIM]_1–*x*_[C_8_MIM-F_13_]_*x*_[Tf_2_N] varies: (a) PNPP and (b) CP and COP.

When the SANS and SAXS data are fitted using a combination of Lorentzian functions, characteristic distances can be extracted. Thus, in Figure 7a, we observe that with an increasing concentration of [C_8_MIM-F_13_][Tf_2_N] in the mixture, the distance represented by the PNPP increases, and for the SANS data, this increase follows a relatively monotonic trend. As such, the smooth change is consistent with intimate mixing of the two components.

Unlike in the SAXS measurements, the COP is not observed in the SANS data. The reason for this is likely to be somewhat complex and cannot be accounted for by contrast matching, as the variation in SLDs for ions of opposite charges is similar in the two techniques. In previous studies of the salts [C_*n*_MIM][Tf_2_N], the COP was observed using SANS for pure ILs when both the ring and chain were deuterated.^6^ For fully hydrogenated samples, the COP was only observed for [C_2_MIM][Tf_2_N]. As hydrogen has a high degree of incoherent scattering, it is very likely that the intensity of the COP was lost into the background for most of these samples.^63^ However, a similar effect would not explain the observations made here, as while [C_8_MIM-*d*_17_][Tf_2_N] is deuterated in the chain only, this still results in a much lower incoherent background than for the fully hydrogenated samples. This background scattering is also relatively low for [C_8_MIM-F_13_][Tf_2_N], as fluorine also has low incoherent scattering.

An alternative interpretation is that the position and intensity of the peaks present in the scattering data are caused by the sum of partial structure factors of different atoms.^64^ This approach leads to peaks and anti-peaks in the partial structure factors which, combined, lead to the observed structure factors. Such features can be seen in the simulations (*vide infra*—Figure 10).

Fitting of the SAXS and SANS data showed variation in the positions of the COP and CP with composition (Figure 7b). The CP moves to longer distances with increasing [C_8_MIM-F_13_] composition and the COP moves to shorter distances, as shown in Figure 7b. When the COP is taken as representing correlations between like charged ions (*e.g.*, cation–anion–cation) and the CP representing anion–cation correlations, it is at first sight surprising that these peaks should change at all as a function of composition since the anions and the cationic headgroups of the two ILs are identical. A better understanding of the origins of the changes seen in the SAXS/SANS data is gained from the complementary atomistic MD simulations that follow.

### MD Simulations of the Bulk

**Figure 8:**
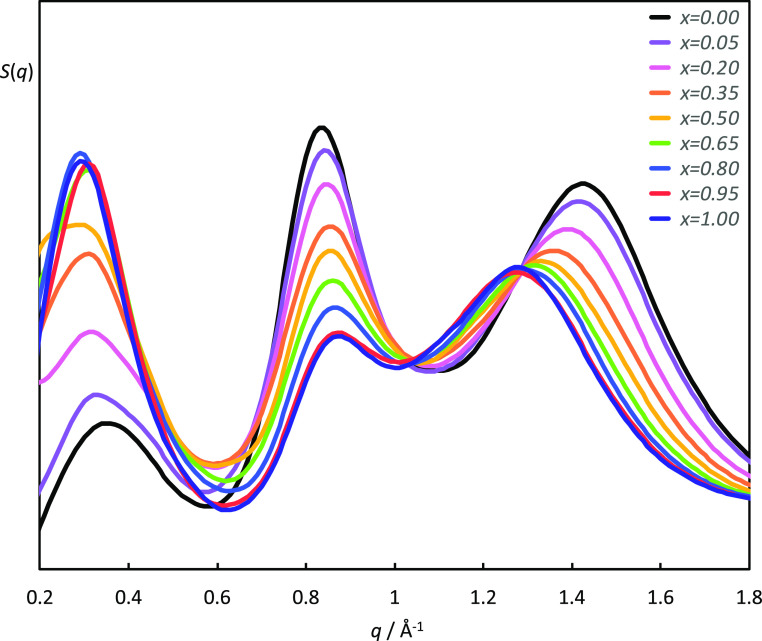
Total structure factor functions, *S*(*q*), [C_8_MIM]_1–*x*_[C_8_MIM-F_13_]_*x*_[Tf_2_N] mixtures at 300 K.

Figure 8 shows the structure factor functions, *S*(*q*), of the [C_8_MIM]_1–*x*_[C_8_MIM-F_13_]_*x*_[Tf_2_N] mixtures, at 300 K, where *x* = 0, 0.05, 0.20, 0.35, 0.50, 0.65, 0.80, 0.95, and 1.00. Since the main interest is in the structural features at the intermolecular level, the analysis focused on the low-*q* regions of the *S*(*q*) functions (0.2 < *q*/Å^–1^ < 1.8) to cover the PNPP, COP, and CP. It was observed that the intensity of the PNPP increases, and it shifts slightly to lower *q*, with the mole fraction of [C_8_MIM-F_13_][Tf_2_N]. At the same time, the intensity of the COP decreases, and its maximum is slightly shifted to higher values of *q*, while the CP shifts to lower *q*-values as *x* increases.

**Figure 9:**
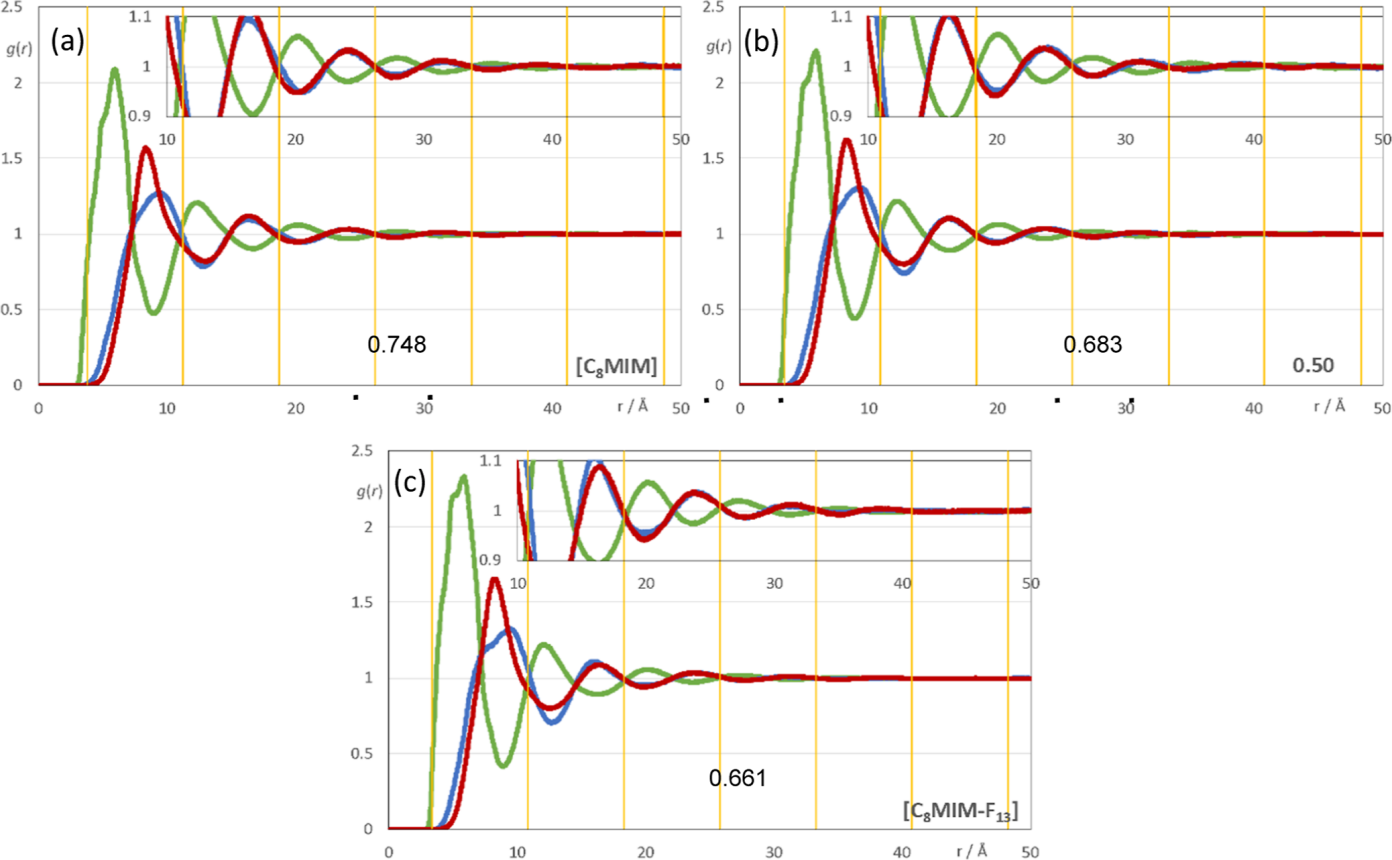
Three selected radial distribution functions (RDFs), *g*(*r*), as a function of distance, *r*, for (a) pure [C_8_MIM][Tf_2_N], (b) pure [C_8_MIM-F_13_]_*x*_[Tf_2_N], and (c) a 1:1 mixture of the two. Green lines: RDFs between selected positions in the cation (imidazolium ring centroid, CM) and anion (nitrogen atom); blue lines: RDFs between imidazolium ring centroids; red lines: RDFs between atoms. The vertical yellow lines reflect the wavelength of the polar domain in each case (number indicated at the bottom) and are related to the position of the corresponding intermediate *q*-peaks in Figure 8. The insets vertically magnify the region between 10 and 50 Å.

The precise *q*-values of those main peaks were calculated by the deconvolution of the corresponding *S*(*q*) functions. The COP *q*-values depend mainly on the characteristic spacing between ions of the same sign separated by a common counterion (Figure 4). In other words, the COP defines the ionic alternation within the polar network mesh that characterizes all ILs. In the present case, the COP slightly shifts to higher *q*-values with the increase in the mole fraction of [C_8_MIM-F_13_][Tf_2_N] and is related to the contraction of the polar network. The mesh value for the polar network that arises from the simulation varies between 2π/0.84 Å^–1^ = 7.5 Å for a pure [C_8_MIM][Tf_2_N] system and 2π/0.87 Å^–1^ = 7.2 Å for pure [C_8_MIM-F_13_][Tf_2_N]. The same general trend can be seen in the experimental data above, although while starting from the same value of 7.5 Å for *x* = 0, the decrease is greater, reaching 6.8 Å at *x* = 1. It must be stressed at this point that the fitting of *S*(*q*) functions at low-*q* with multiple complicated functions was performed taking into account the position of the COP peak for each mixture. Thus, a very strong link is observed between the position of the COP and the correlation functions, *g*(*r*), between pairs of ions with the same or opposite charges. In order then to avoid consistency problems associated with multi-peak fitting redundancies, a check was always made to ensure that the characteristic wavelength of the charge-ordering *g*(*r*) functions of a given system and the position of the fitted COP peak were consistent. This is shown in Figure 9a–c, where the corresponding charge-ordering *g*(*r*) functions are depicted. A grid representing the characteristic wavelength for each system (2π/*q*), obtained from the fitted COP values, is superimposed with the correlation functions and confirms the consistency of the fitting methodology. The deconvolution results are listed in Table 2.

**Table 2:** Position (*q*-Values, Å^–1^, and Spacings, Å) of the Gaussian Curves Used in the Deconvolution of the MD *S*(*q*) Functions

*X*_[C_8_MIM-F_13_][Tf_2_N]_	PNPP/Å^–1^ (Å)	COP (from RDFs)/Å^–1^ (Å)	CP/Å^–1^ (Å)
0.00	0.363 (17.31)	0.842 (7.46)	1.405 (4.47)
0.05	0.352 (17.85)	0.846 (7.43)	1.398 (4.49)
0.20	0.320 (19.63)	0.851 (7.38)	1.374 (4.57)
0.35	0.304 (20.67)	0.856 (7.34)	1.348 (4.66)
0.50	0.275 (22.85)	0.857 (7.33)	1.320 (4.76)
0.65	0.300 (20.94)	0.861 (7.30)	1.301 (4.83)
0.80	0.294 (21.37)	0.866 (7.26)	1.284 (4.89)
0.95	0.308 (20.40)	0.866 (7.26)	1.262 (4.98)
1.00	0.299 (21.01)	0.869 (7.23)	1.262 (4.98)

**Figure 10:**
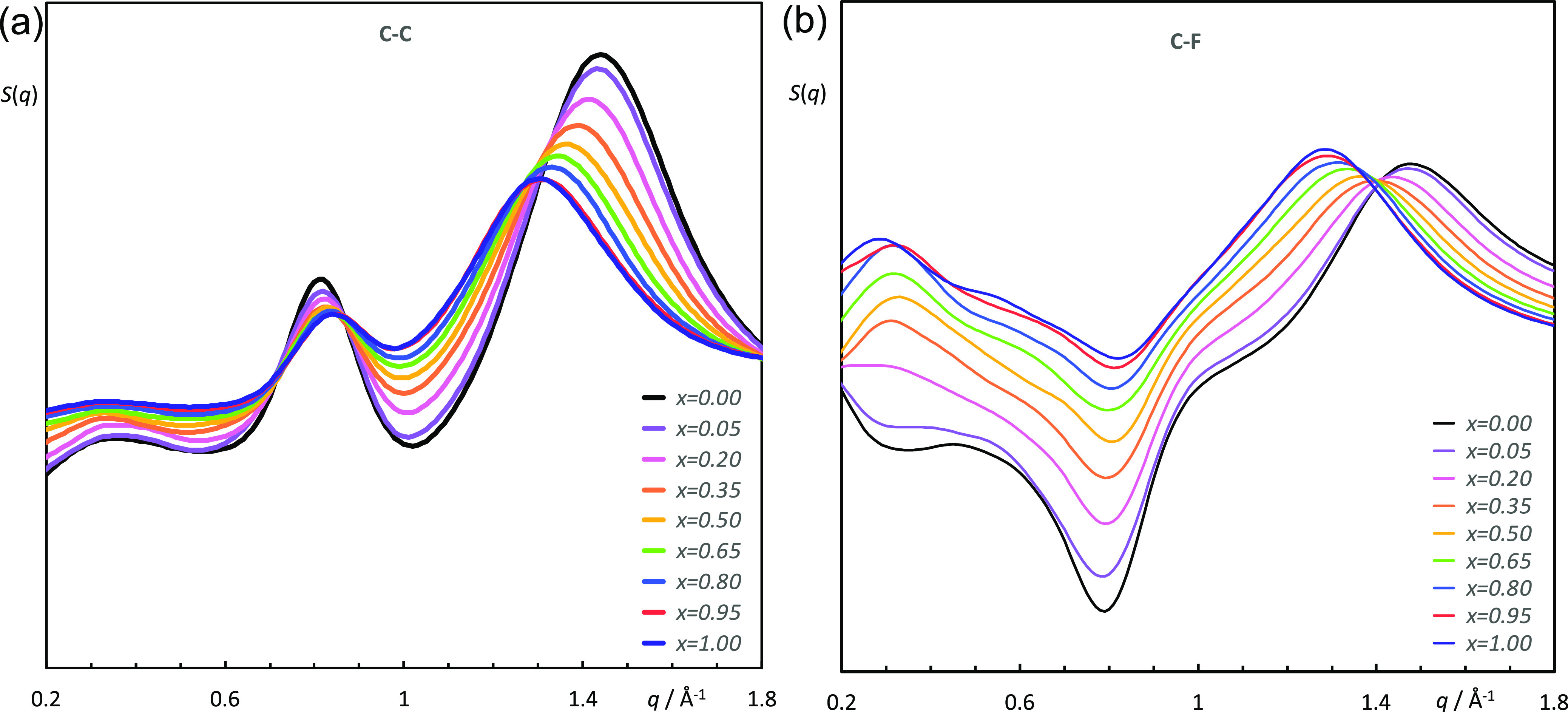
Two representative partial structure factor functions, *S*(*q*), of the [C_8_MIM]_1–*x*_[C_8_MIM-F_13_]_*x*_[Tf_2_N] mixtures corresponding to (a) C···C and (b) C···F pair correlations.

The CP is the result of multiple contributions from different distance correlations, which means that it is difficult to assign its *q*-value shifts to specific intermolecular arrangements. Indeed, its value is normally associated with the nature of the ions and their corresponding intermolecular contact distances (Figure 4), broadly defining the boundary between intra- and inter-molecular structural features and being a general structural feature present in most ILs (and molecular fluids). Increasing the mole fraction of [C_8_MIM-F_13_][Tf_2_N] in these mixtures causes the CP to shift to lower *q*-values, which is explained by considering the partial structure factors shown in Figure 10a,b. These data, which match well those from the experiment, suggest that one of the main contributors to the CP can be assigned to intermolecular C···C and C···F correlations, so that as the mole fraction of [C_8_MIM-F_13_][Tf_2_N] increases, the semiperfluoro chains dominate and so the longer C–F bonds (compared to C–H) lead to an increase in the C···F and C···C distances within the chain regions of the IL, consistent with the observed trends.

The calculated COP *q*-values listed in Table 2 show the same trend (shift to higher *q*-values) detected experimentally. This is somewhat surprising since COP values tend to shift to lower *q*-values as the volume occupied by the non-polar alkyl side chains increases and expands the polar network. This is the situation observed both experimentally and by simulation for the [C_*n*_MIM][Tf_2_N] system (2 < *n* < 10) and in the IL mixture system [C_*2*_MIM]_1–*x*_[C_12_MIM]_*x*_[Tf_2_N],^55,65^ and one might, therefore, expect a similar trend in the present systems, as the hydrogenated side chains are replaced by their bulkier fluorinated counterparts. However, it is found (Figure 9a,c) that the interionic distance between two anions separated by a cationic imidazolium ring bearing a fluorinated chain is shorter than the corresponding distance where the chain on the cation is hydrogenous.

**Figure 11:**
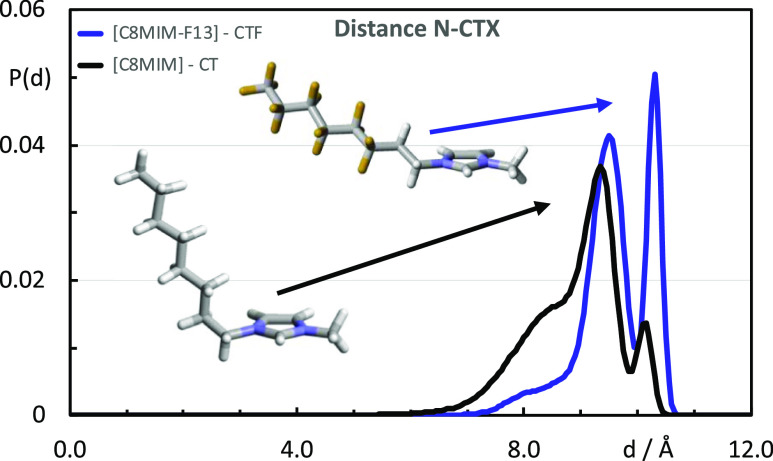
Probability distribution functions, *P*(*d*), of the intramolecular distances between the N atom of the imidazolium ring and the terminal carbon atoms of the alkyl chain in [C_8_MIM][Tf_2_N] (black line) and [C_8_MIM-F_13_][Tf_2_N] (blue line).

To help understand this, the distribution of distances between the nitrogen of the imidazolium ring attached directly to the alkyl side chain and the last carbon atom of that chain (imidazole chain terminus distances) in the two cations (orange and red colors, respectively) are plotted in Figure 11. Each distribution shows two peaks and a shoulder. The peak at longer distances corresponds to alkyl chains in their fully extended, anti-conformation, whereas the peak at shorter distances corresponds to otherwise fully extended chains but with a gauche conformation at the C1–C2 bond (*cf.*Figure 11, inset). The shoulder, which corresponds to shorter distances, indicates that some chains may contain additional gauche conformations. The two distribution functions confirm that the more rigid fluorinated chains prefer all-anti conformations, whereas the more flexible hydrogenated chains generally “bend” at the C1–C2 bond and may also display additional kinks in the chain.^66^ As such, while these gauche conformations may lead to some shortening of the effective length of the chain, the effective volume that results in terms of lateral packing increases, consistent with the greater spacing observed for the COP. In contrast, and despite the greater overall volume of the fluorocarbon chain, the preferred all-trans conformation of the [C_8_MIM-F_13_]^+^ cation is consistent with a more effective “packing” in the liquids containing larger proportions of [C_8_MIM-F_13_][Tf_2_N], leading to a decrease in inter-ionic separation and the observation of the COP moving to higher *q*-values.

**Figure 12:**
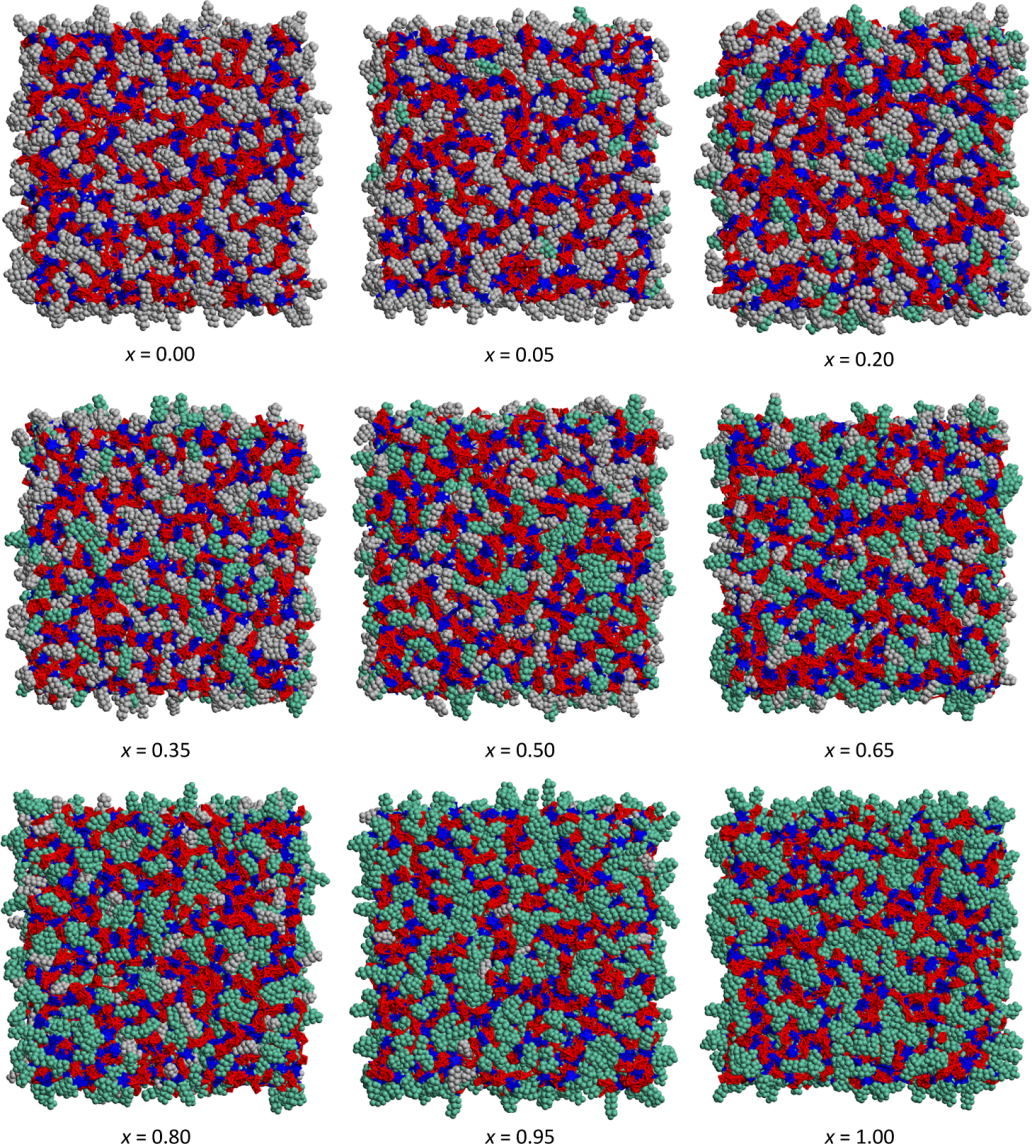
MD simulation snapshots illustrating the nanosegregation between the polar network (red/blue mesh) and non-polar domains (gray-green beads) in [C_8_MIM]_1–*x*_[C_8_MIM-F_13_]_*x*_[Tf_2_N] mixtures.

**Figure 13:**
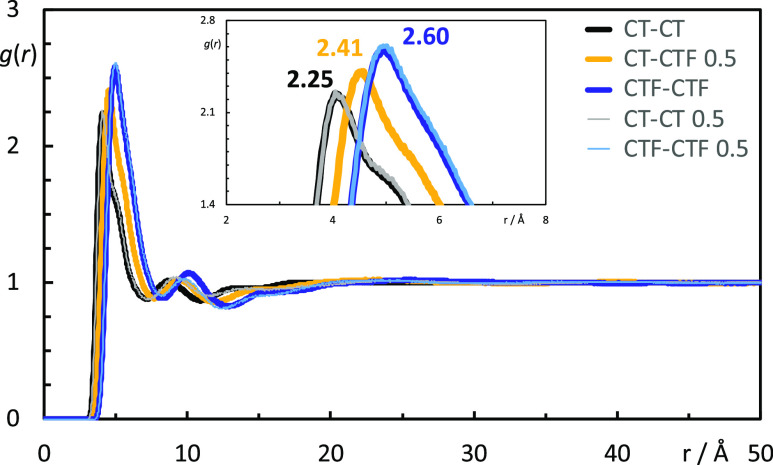
Five selected radial distribution functions (RDFs), *g*(*r*), as a function of distance, *r*, for three selected [C_8_MIM]_1–*x*_[C_8_MIM-F_13_]_*x*_[Tf_2_N] mixtures, *x* = 0, 0.50, and 1.00. Black lines: RDFs between the terminal carbon atoms of [C_8_MIM], CT; blue lines: RDFs between the terminal carbon atoms of [C_8_MIM-F_13_], CTF; orange line: RDFs between the terminal carbon atoms of [C_8_MIM] and the terminal carbon atoms of [C_8_MIM-F_13_]. The insets magnify the peak region.

The PNPP *q*-values listed in Table 2 reflect characteristic distances around 20 Å that are typical of the localized segregation between polar and non-polar domains in ILs with octyl side chains.^32^ This self-organized structure of [C_8_MIM]_1–*x*_[C_8_MIM-F_13_]_*x*_[Tf_2_N] mixtures can be summarized visually in the MD simulation snapshots presented in Figure 12. These snapshots represent all of the mixtures studied by MD simulations, and in them, it is possible to observe that both alkyl and perfluoroalkyl chains are close together, with no apparent segregation. This lack of segregation can be quantified by analyzing the pair radial distribution functions between the terminal carbon atoms of the alkyl chains (CT and CTF for the hydrogenated and fluorinated chains, respectively). Figure 13 shows such comparison and confirms that the probability of finding a “mixed” CT–CTF pair is the simple average of the probabilities of finding “pure” CT–CT or CTF–CTF pairs. The same trend was observed by Kirchner *et al.* for mixtures of [C_8_MIM]Br and [C_8_MIM-F_13_]Br.^37^

In some cases (namely, for mixtures with a higher fraction of fluorinated cations), the deconvolution of the *S*(*q*) functions in the PNPP region can be better achieved using two Gaussian curves. The main Gaussian curve, corresponding to a *q*-value of ≈0.3 Å^–1^ (20 Å characteristic distance), is superimposed with a much smaller Gaussian curve at ≈0.45 Å^–1^ (14 Å characteristic distance). The existence of a “shoulder” associated with the PNPP has been observed in other systems containing fluorinated alkyl side chains,^37,67^ and it was suggested that this could be evidence of segregation between the fluorinated and hydrogenated domains. However, the “shoulder” in this case occurs to higher *q* than the PNPP, whereas segregation of the fluorous and aliphatic regions of the IL would be expected to lead to scattering at lower *q* (longer length scales).^68^ In addition, since the PNPP is also best described by two Gaussian curves in the pure fluorinated system (where no segregation can occur), one must conclude that, at least in this case, the existence of a small shoulder at higher *q* is caused by the presence of fluorine in the non-polar domains and is not, therefore, evidence of segregation between the different chain types.

## Discussion and Conclusions

*Inter alia*, this study highlights the insight that may be gained by using SAXS and SANS as complementary structural probes supported by some of the more quantitative insights available from MD and with complementary physical property measurements.

The separation represented by the PNPP increases from *ca.* 19 Å [C_8_MIM][Tf_2_N] to *ca.* 21 Å for pure [C_8_MIM-F_13_][Tf_2_N], which can be seen experimentally in SANS and simulation and could appear to be simply rationalized by the greater volume and rigidity of the fluorocarbon chains compared to alkyl chains. However, the reality is a little more subtle. Thus, the flexibility associated with the hydrocarbon chain, which can result in chain folding, is absent in perfluorinated chains, so that the only flexibility arises from the dimethylene spacer between the imidazole ring and the perfluorocarbon segment. As such, the length of the bilayer is more likely to reflect the fully extended length of the fluorinated chain. Furthermore, the MD simulations show that while in the fluorous cations there is an approximately equal population of cations with two trans C–C bonds and with one trans and one gauche, in the hydrogenous cations, the conformer population is dominated by cations with one trans and one gauche C–C bonds, with an appreciable population of cations with two gauche C–C bonds. This is likely an expression of the observation that hydrocarbon chains tend to coil when dissolved in (*i.e.*, mixed with) fluorocarbons.^69^ Thus, as shown in Figure 11, gauche C–C bonds shorten the distance from the imidazolium ring to the terminal chain carbon, and so, evidently conformational factors also contribute to the evolution of the PNPP with mixture composition. Finally, on this point, the observation of a monotonic increase in the magnitude of the PNPP in the mixtures [C_8_MIM]_1–*x*_[C_8_MIM-F_13_]_*x*_[Tf_2_N] suggests that neither component dominates the structure. Interestingly, this contrasts with previous observations for the mixtures [C_12_MIM]_*x*_[C_8_MIM]_1–*x*_[Tf_2_N], discussed in the Introduction section, where a non-linear evolution of the PNPP distance was found, indicating that the structure of the [C_12_MIM][Tf_2_N] IL dominated in the mixtures.^15^

Both the experiment and simulation also show an increase in the CP distance as the proportion of [C_8_MIM-F_13_][Tf_2_N] in the mixtures increases, and the calculated correlation functions show that this is driven by the greater spacing between the fluorocarbon chains attached to the cation compared to the hydrocarbon chains. As such, the CP is not simply a reflection of nearest-neighbor cation···anion distances but rather also reflects the spacing of any attached chains. Where such chains are hydrocarbon in nature, then the chain···chain and cation···anion spacings are very similar and so will not normally be resolved where the peak is broad. Indeed, this is the approach in the structural study of liquid crystals where the broad, wide-angle peak at *q* ≈ 1.36 Å^–1^ is attributed to the spacing between molten alkyl chains and, when the chains are fluorocarbon in nature, the peak moves to smaller *q* (≈1.15 Å^–1^) to reflect the greater size of the fluorocarbon chains.^70^

Curiously, while the CP moves to lower *q* as the proportion of [C_8_MIM-F_13_][Tf_2_N] in the mixtures increases, the COP moves in the opposite direction, with a tightening of the polar mesh. While at first sight counterintuitive, consideration of the conformational preferences of the chains with respect to the cation ring shows a preference for an extended arrangement of the fluorocarbon chains that allows more efficient packing of the ions and can support the tighter mesh. This contrasts with the presence of more gauche bonds in the hydrocarbon chains, which leads to an increase in their effective volume and so acts to push them apart.

Hydrocarbons and fluorocarbons are recognized as generally immiscible and with this observation comes the notion of a fluorous or fluorophobic effect. This is normally associated with longer perfluorocarbon chain segments, and the idea likely originates in the way the critical solution temperature of mixtures of C_*n*_H_2*n*+2_ with C_*n*_F_2*n*+2_ increases with *n*, from −40 °C (*n* = 4) through 23 °C (*n* = 6) to 76 °C (*n* = 8)—*i.e.* room-temperature immiscibility is more likely at longer chain lengths.^71^ Recent consideration of the origin of the immiscibility using computational methods has proposed that weak intermolecular interactions between fluorocarbons and hydrocarbons have their origin in the unfavorable ground-state geometries of the former which, on account of the much greater rigidity of these chains, do not easily distort. Furthermore, the inaccessibility of other geometries also modulates inter-chain interactions in pure fluorocarbons, accounting for their weaker intermolecular interactions.^35^

In considering the organization in these materials, it is then important to ponder on the extent to which the data indicate any fluorous-driven phase separation and the formation of a triphilic arrangement. Thus, triphilic organization appears evident from experimental and computational studies in ILs where the cation is hydrogenous and the anion is fluorous,^72,73^ and there is evidence for such organization in both the liquid crystal phase and the isotropic liquid phase of some molecularly triphilic triazolium salts.^68^ Further, Hollóczki *et al.*(37) undertook a computational investigation of mixtures of the same cations studied here but with a bromide anion in place of bistriflimide, proposing the existence of triphilic organization from a calculated structure factor to higher-*q* from the main PNPP.

**Figure 14:**
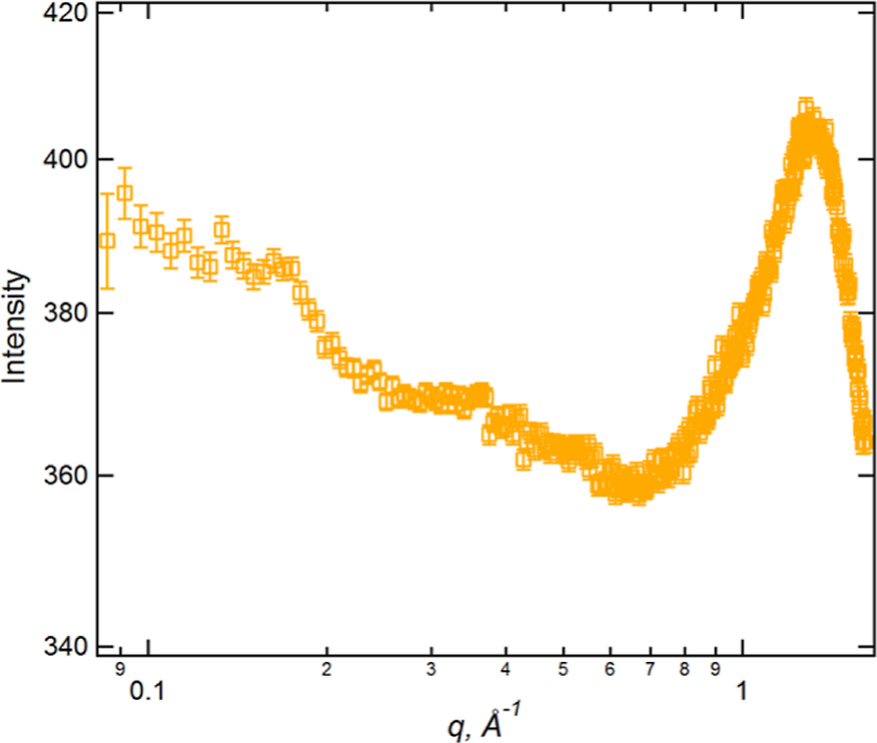
SANS data, acquired on D16, for [C_8_MIM][Tf_2_N]_0.5_[C_8_MIM-F_13_]_0.5_[Tf_2_N] showing the presence of a low-intensity peak at *q* = 0.16 Å^–1^.

From the data collected for the PNPP at beamline D16 at the ILL, from SANS2D at ISIS, as well as the related SAXS data, it can be inferred that the hydrocarbon and fluorocarbon chains in [C_8_MIM]_1–*x*_[C_8_MIM-F_13_]_*x*_[Tf_2_N] are both located within a single non-polar domain. This arises first from the observation of a smooth evolution of the PNPP with composition and also the observation that the probability of finding a “mixed” CT–CTF pair is the simple average of the probabilities of finding “pure” CT–CT or CTF–CTF pairs. Furthermore, while the PNPP can be deconvoluted into a smaller- and larger-*q* Gaussian curve, this is true in both pure [C_8_MIM][Tf_2_N] and pure [C_8_MIM-F_13_][Tf_2_N]. As such, it cannot arise from greater segregation into a triphilic arrangement, and indeed, the additional Gaussian is to greater *q* than that of the main peak, which would not be expected as an expression of additional local order arising from segregation. That said, there is a weak feature observed to lower *q* than the PNPP in the SANS data acquired for [C_8_MIM]_0.5_[C_8_MIM-F_13_]_0.5_[Tf_2_N] on D16 (Figure 14), whose length scale might be more reflective of triphilic organization.^a^ Furthermore, the fact that it is weak means that regardless of its origin, it may not impinge at all strongly on any of the other indicators we have proposed to suggest the general miscibility of the alkyl and fluoroalkyl chains within these mixtures.

In the present system, the electrostatic attraction through the imidazolium/bistriflimide pairing may be sufficient to prevent phase separation, a proposal that is consistent with almost all of the data to hand. However, the possibility may remain for some small, local domain formation not observed through the MD simulations, possibly indicated in the D16 data, at some compositions. As such, the conclusion is drawn that the chain lengths alone are insufficient to drive any appreciable phase separation, and so, it remains to be seen how this behavior will evolve as the chains are extended further. Experiments of this type are in progress.

## Supporting Information Available

Synthetic and instrumental methods, density data, viscosity data and plots, and fitting parameters used for processing SAXS and SANS data (PDF)

## Footnotes

a Owing to the different ways in which data are collected at D16 and SANS2D, this feature cannot be seen in the data obtained on the latter beamline (see Figure S7).
