# Supplementary material for: Understanding the Liquid Structure in Mixtures of Ionic Liquids with Semiperfluoroalkyl or Alkyl Chains
Source: J Phys Chem B. 2023 Aug 9;127(33):7394–407. doi: 10.1021/acs.jpcb.3c02647 (PMC10461228; doi:10.1021/acs.jpcb.3c02647)
Supplement: Supplementary file 1 — jp3c02647_si_001.pdf [file jp3c02647_si_001.pdf]

Understanding Liquid Structure in Mixtures of Ionic Liquids with Semiperfluoroalkyl or Alkyl Chains

Naomi S. Elstone[a], Karina Shimizu*[b], Emily V. Shaw[a], Paul D. Lane[c], Lucía D'Andrea[a], Bruno Demé[d], Najet Mahmoudi[e], Sarah E. Rogers[e], Sarah Youngs[e], Matthew L. Costen[c], Kenneth G. McKendrick[c], Jose N. Canongia Lopes[b], Duncan W. Bruce*[a] and John M. Slattery*[a]

[a] Department of Chemistry, University of York, Heslington, York YO10 5DD, U.K. duncan.bruce@york.ac.uk; john.slattery@york.ac.uk.

[b] Centro de Química Estrutural, Institute of Molecular Sciences, Instituto Superior Técnico, Universidade de Lisboa, Av. Rovisco Pais 1049 001 Lisboa, Portugal. karina.shimizu@tecnico.ulisboa.pt

[c] Institute of Chemical Sciences, School of Engineering and Physical Sciences, Heriot-Watt University, Edinburgh, EH14 4AS, U.K.

[d] Institut Laue-Langevin, 38000 Grenoble, France.

[e] ISIS Neutron Source Facility, Harwell Science and Innovation Campus, Didcot, OX11 0DE, UK.

Supplementary Information

Synthetic methodologies

Air-sensitive experimental procedures were carried out under an inert nitrogen atmosphere using standard Schlenk line and glovebox techniques. Acetonitrile, hexane and toluene were purified using an Innovative Technologies anhydrous solvent engineering system. 1-methylimidazole was dried over calcium hydride and vacuum distilled. 1-Bromooctane was dried over activated molecular sieves and distilled immediately prior to use. Deuterated [D_{17}]-1-bromooctane was provided by the Isis deuteration facility and used without further purification. ^{1}H and ^{19}F NMR spectra were acquired at 293 K on a JEOL ECX-400.

Synthesis [C_8MIM]Br

This product was prepared according to the literature method.[1] 1-Methyl imidazole (64 mL, 0.8 mol) was added to a solution of freshly distilled 1-bromooctane (148.25g, 0.77 mol) in dry toluene (150 mL) and heated to 60 °C under an inert atmosphere overnight. Toluene was removed with a syringe and under vacuum at elevated temperatures for 7 days. Some excess 1-methylimidazole remained, this is removed in the metathesis (198.54g, 96% yield). ^{1}H NMR (400 MHz, acetone-d6, 293 K), δ (ppm): 10.23 (s, 1H), 7.94 (m, 1H), 7.87 (m, 1H), 4.45 (t, 2H), 4.10 (s, 3H), 1.94 (quint ,2H), 1.29 (m, 10H), 0.86 (t, 3H)

Synthesis [C_8MIM][Tf_2N]

The synthetic route to this product was adapted from the literature method.[1] [C_8MIM]Br (50 g, 0.18 mol) in dichloromethane (300 mL) was added to a solution of Li[Tf_2N] (68 g, 0.24 mol) in deionised water and stirred for two days at room temperature. The DCM layer, containing the IL, was separated and washed (3 x 200 mL) deionised water. Two subsequent negative tests for halide with $AgNO_3$ were required to ensure removal of LiBr. The DCM was removed using rotary evaporation to leave the IL and this further was dried under vacuum at 60 °C. The NMR indicated that all DCM had been removed (78.22 g, yield: 92%). Purity was checked using elemental analysis.
^{1}H NMR (400 mHz, *acetone-d$_6$,* 293 K), δ (ppm): 8.94 (s, 1H), 7.71 (m, 1H), 7.65 (m, 1H), 4.33 (t, 2H), 4.01 (s, 3H), 1.91 (quint, 2H), 1.29 (m, 10H), 0.85 (t, 3H)
Elemental analysis: %C: 35.2 (calc 35.37), %H 4.75 (calc 4.88), %N: 8.71 (calc 8.63)
Water content: 614 ppm

Synthesis of [C_8MIM-d_{17}][Tf_2N]

The same procedure described above was used to prepare the deuterated compound. [D_{17}]-1-bromooctane (12g, 0.051 mol) was reacted with 1-methylimidazole (4.4mL, 0.055 mol) in dry toluene (120 mL). [D_{17}-C_8MIM]Br (10.16 g, 0.035 mol) was dissolved in DCM and added to a solution of Li[Tf_2N](10.97g, 0.038 mol) in deionised water. IL Purity was checked using elemental analysis and the degree of deuteration was determined use ^{1}H NMR (15.14g, 60% yield).
^{1}H NMR (400 mHz, *acetone-d$_6$,* 293 K), δ (ppm): 8.96 (s, 1H), 7.71 (m, 1H), 7.61 (m, 1H), 4.01 (s, 3H), degree of deuteration>97%
Elemental analysis: %C 34.23 (34.13), %H 8.10(8.65), %N 8.24 (8.33)

Synthesis [C_8MIM-F_{13}]I

This product was prepared according to the literature method.[1] 1-Methylimidazole (7 mL, 7.21 g, 0.088 mol) was added to a solution of *1H,1H,2H,2H*-perfluorooctyl iodide (23.5 mL,45.5 g, 0.096 mol) in acetonitrile. The resulting solution was heated to 60 °C and stirred under an inert N_2 atmosphere

for 3 weeks, with progression monitored using ^1H and ^{19}F NMR. The acetonitrile was removed under vacuum. The resulting solid was washed with dry hexane (50 mL x 3) to removes excess *1H,1H,2H,2H*-perfluorooctyl iodide. The solid was dried under vacuum (35.22g, 72% yield). Remaining impurities are removed during metathesis.

^1H NMR (400 mHz, *acetone-d$_6$,* 293 K), δ (ppm): 9.71 (s, 1H), 8.03 (m, 1H), 7.84 (m, 1H), 4.92 (t, 2H), 4.10 (s, 3H), 1.94 (quint ,2H), 1.29 (m, 10H), 0.86 (t, 3H)
^{19}F NMR (376 MHz, *acetone-d$_6$,* 293 K) δ (ppm): -81.5 (3F), -114.3 (2F), -122.3 (2F), -123.3 (2F), -123.9 (2F), -126.7 (2F)

Synthesis [C_8MIM-F_{13}][Tf_2N]

The synthetic route to this product was adapted from the literature method.[1]The solid [C_8MIM-F_{13}]I (34.72g, 0.062 mol) was dissolved in DCM and added to a solution of Li[Tf_2N] (20.83g, 0.073 mol) in deionised water and stirred at room temperature for 2 days. The DCM layer, containing the IL, was separated and washed (5 x 200 mL) deionised water. Two subsequent negative tests for halide with $AgNO_3$ were required to ensure removal of LiI. DCM was removed using rotary evaporation and the IL was dried under vacuum at 60°C to afford a dark amber oil (26.80g, 61% yield). Purity of the compound was checked by elemental analysis.
^1H NMR (400 mHz, *acetone-d$_6$,* 293 K), δ (ppm): 9.12 (s, 1H), 7.88 (m, 1H), 7.72 (m, 1H), 4.81 (t, 2H), 4.04 (s, 3H), 3.11 (m, 2H)
^{19}F NMR (376 MHz, *acetone-d$_6$,* 293 K) δ (ppm):-79.9 (6F), -81.7 (3F), -114.6 (2F), -122.4 (2F), -123.5 (2F), -124.2 (2F), -126.9(2F)
Comparison of NMR analysis with existing data are consistent and within error when the same solvent[1] is used and comparable when a different solvent[2] was selected.
Elemental analysis: %C 23.58 (23.70), %H 1.28 (1.42), %N 5.54 (5.93)
Water content: 271 ppm

Density Data

Table S1: Density, excess density, molar volume and excess molar volume data at 293.15 K for the [C_8MIM]$_{1-x}$[C_8MIM-F_{13}]$_x$[Tf_2N] IL mixtures

x	M/ g mol^{-1}	*ρ* / g cm^{-3}	Δ *ρ* / %	V_m / cm^{3}mol^{-1}	Δ V_m / %
0.00	475.47	1.325277	-	358.7702	-
0.05	487.1635	1.347324	0.105575	361.5787	-0.10546
0.20	522.244	1.412483	0.334127	369.7348	-0.33301
0.35	557.3245	1.473836	0.284506	378.1454	-0.2837
0.50	592.405	1.535697	0.271988	385.7565	-0.27125
0.65	627.4855	1.594508	0.069081	393.5292	-0.06903
0.80	662.566	1.652562	-0.16444	400.9327	0.16471
0.95	697.6465	1.709047	-0.47243	408.2078	0.474676
1.00	709.34	1.737785	-	408.1862	-

Viscosity Data

Table S2: Viscosity data for the IL mixture $[C_3MIM]_{1-x}[C_8MIM\text{-}F_{13}]_x[Tf_2N]$ in the temperature range between 298 and 328 K

x	Viscosity, mPa s						
	T,K						
	**298**	**303**	**308**	**313**	**318**	**323**	**328**
0.00	85.54	68.07	54.11	43.77	35.89	29.22	24.67
0.05	99.51	76.67	60.69	48.74	39.77	32.13	27.22
0.20	137.41	103.89	81.04	64.06	51.58	41.17	34.43
0.35	187.85	142.90	109.71	85.94	68.17	53.80	44.52
0.50	260.12	192.65	145.24	111.97	87.20	67.33	55.78
0.65	385.08	278.48	205.81	155.52	119.52	91.03	73.30
0.80	579.21	412.79	300.60	223.97	169.62	127.83	102.56
0.95	909.53	626.16	444.07	321.86	238.00	174.99	137.34
1.00	1049.45	716.68	504.45	364.17	268.47	195.98	153.04

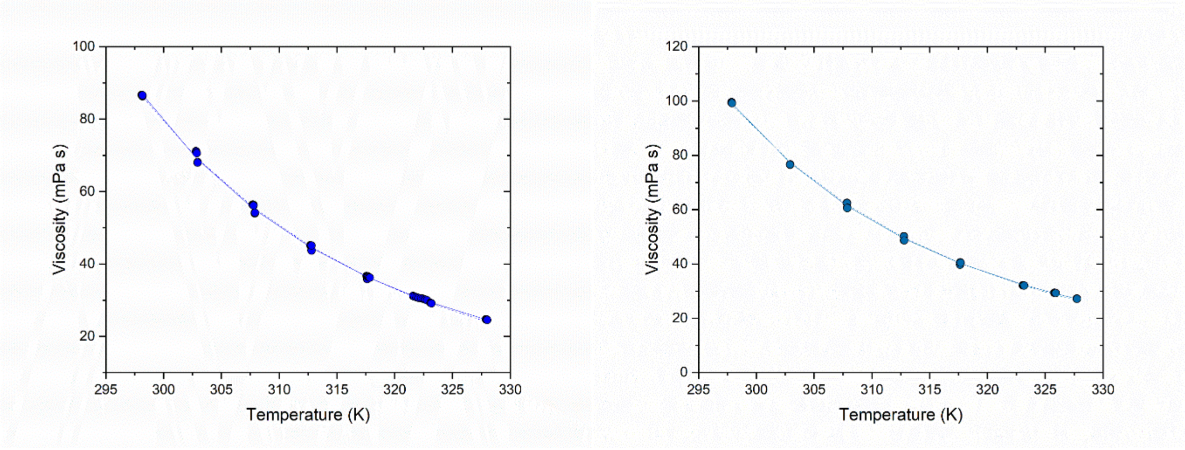

Figure S1: Temperature dependence of the viscosity (η) of neat (left) $[C_8MIM][Tf_2N]$ and (right) $[C_8MIM]_{0.95}[C_8MIM\text{-}F_{13}]_{0.05}$ $[Tf_2N]$ Experimental values are represented by the markers, the dashed line indicates the Arrhenius model and the solid line indicates the VFT model.

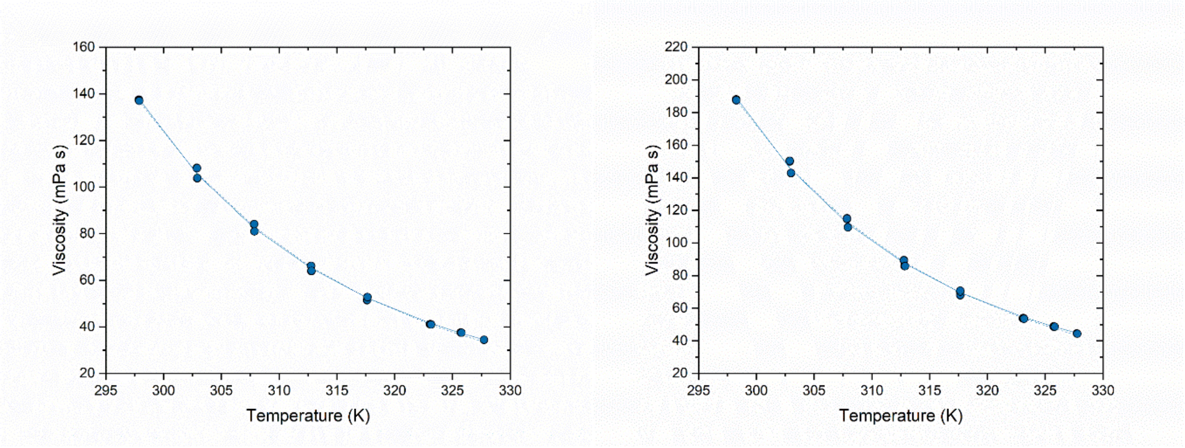

Figure S2: Temperature dependence of the viscosity (η) of (left) $[C_8MIM]_{0.8}[C_8MIM\text{-}F_{13}]_{0.2}[Tf_2N]$ and (right) $[C_8MIM]_{0.65}$ $[C_8MIM\text{-}F_{13}]_{0.35}[Tf_2N]$. Experimental values are represented by the markers, the dashed line indicates the Arrhenius model and the solid line indicates the VFT model.

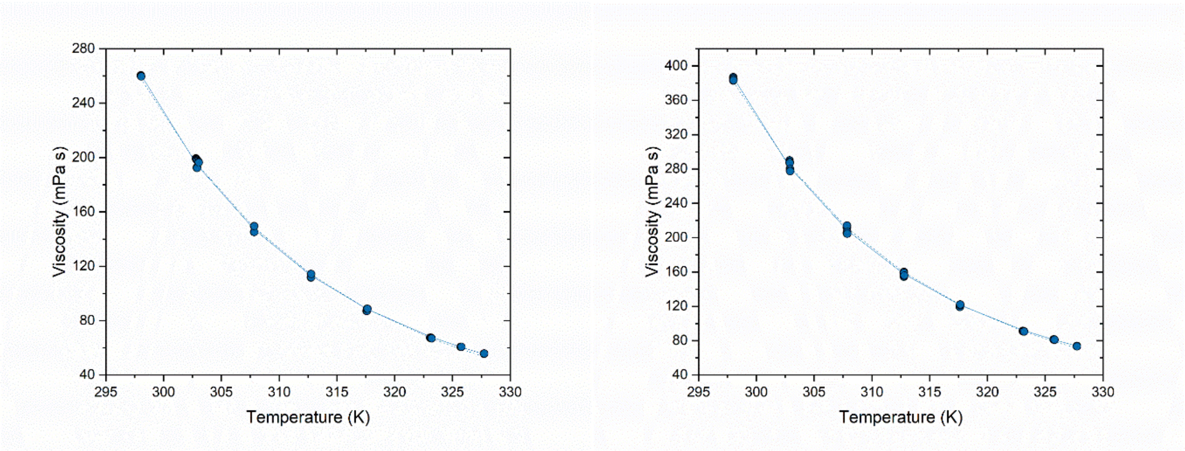

Figure S3: Temperature dependence of the viscosity (η) of (left) $[C_8MIM]_{0.5}[C_8MIM\text{-}F_{13}]_{0.5}[Tf_2N]$ and (right) $[C_8MIM]_{0.35}[C_8MIM\text{-}F_{13}]_{0.65}[Tf_2N]$. Experimental values are represented by the markers, the dashed line indicates the Arrhenius model and the solid line indicates the VFT model.

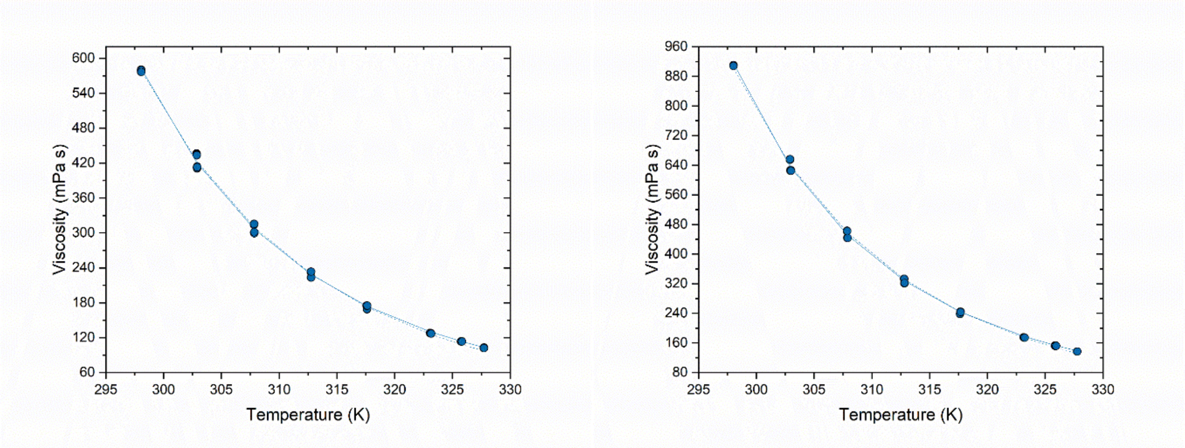

Figure S4: Temperature dependence of the viscosity (η) of (left) $[C_8MIM]_{0.2}[C_8MIM\text{-}F_{13}]_{0.8}[Tf_2N]$ and (right) $[C_8MIM]_{0.05}[C_8MIM\text{-}F_{13}]_{0.95}[Tf_2N]$. Experimental values are represented by the markers, the dashed line indicates the Arrhenius model and the solid line indicates the VFT model.

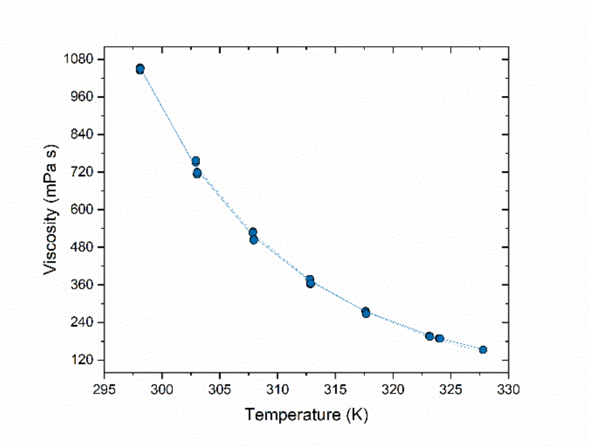

Figure S5: Temperature dependence of the viscosity (η) of neat $[C_8MIM\text{-}F_{13}][Tf_2N]$ Experimental values are represented by the markers, the dashed line indicates the Arrhenius model and the solid line indicates the VFT model.

Figures S1 to S15 show the measured viscosity data as a function of temperature. The temperature dependence of each ionic liquid mixture was fitted by the Vogel-Fulcher-Tammann equation (VFT):

$$log_{10}\eta_x = log_{10}A_\eta + \frac{k_\eta}{T - T_0}$$

where ηx is the viscosity (mPa s) of the mixture with bulk mole fraction *x*, A_η (mPa s), k_η, and T_o are fitting constants with $k_{\eta,}$ and T_o having units of Kelvin.

SAXS

Fit parameters

Table S1: Fit parameters used to fit the SAXS data using a combination of Lorentzian peaks and an Ornstein-Zernicke model to determine correlation length (CL)

x	PNPP Å^{-1}	HWHM Å^{-1}	COP Å^{-1}	HWHM Å^{-1}	CP Å^{-1}	HWHM, Å^{-1}	CL Å
**0.00**	0.35 ±0.02	0.17 ± 0.01	0.85 ± 0.02	0.21± 0.08	1.33 ± 0.03	0.26 ±0.03	-
**0.05**	0.31 ±0.01	0.23 ± 0.01	0.85 ± 0.01	0.18 ± 0.03	1.32 ± 0.01	0.31 ±0.04	3.9 ± 0.07
**0.20**	0.31 ±0.01	0.24 ± 0.01	0.87 ± 0.01	0.19 ± 0.01	1.31 ± 0.01	0.30 ± 0.02	-
**0.35**	0.28 ± 0.01	0.28 ± 0.01	0.88 ± 0.02	0.18 ± 0.01	1.29 ± 0.02	0.29 ± 0.02	-
**0.50**	0.32 ± 0.05	0.30 ± 0.05	0.89 ± 0.02	0.16 ± 0.01	1.27 ± 0.02	0.22 ± 0.04	4.2 ± 0.7
**0.65**	-	-	0.92 ± 0.02	0.14 ± 0.01	1.26 ± 0.07	0.29 ± 0.01	1.4 ± 0.04
**0.80**	0.32 ± 0.03	0.39 ± 0.04	0.92 ± 0.02	0.15 ± 0.02	1.23 ± 0.02	0.29 ± 0.01	1.2 ± 0.1
**0.95**	0.30 ± 0.03	0.55 ± 0.7	0.93 ± 0.03	0.13 ± 0.03	1.22 ± 0.04	0.33± 0.01	-
**1.00**	0.39 ± 0.02	0.39 ± 0.02	0.94 ± 0.02	0.17 ± 0.01	1.20 ± 0.03	0.27 ± 0.01	-

In addition, some additional experiments were conducted using the DL-SAXS instrument at Diamond, allowing access to scattering at smaller *q*, but the data (DOI: 10.15124/31b8a7ba-8465-4e92-a114-1101207b183d) did not yield any information not already in hand from the in-house instrument.

SANS

D16 ILL: Grenoble, France

SANS was carried at the Institut Laue-Langevin (ILL, Grenoble, France) on the D16 instrument, a cold neutron diffractometer, which uses a highly orientated pyrolytic graphite monochromator to focus the beam along the vertical axis. This gave access to a neutron wavelength of 4.47 Å. The nine crystals which make up the monochromator are orientated to maximise the incident neutron flux by focussing the beam to the sample. The detector angle γ was varied (13, 29, 45 and 61°) in order to access the desired *q* range, with the samples tilted 10° away from the detector in the Z axis. The distance of the MWPC detector relative to the sample was fixed, at 950mm from the sample. This 2D ^{3}He detector (MILAND) is made of 320 X by 320 Y wires spaced by 1 mm providing a pixel resolution of 1 mm × 1mm and a detection area of 320 × 320 mm. The resulting *q* range was 0.085-1.6 Å^{-1}.

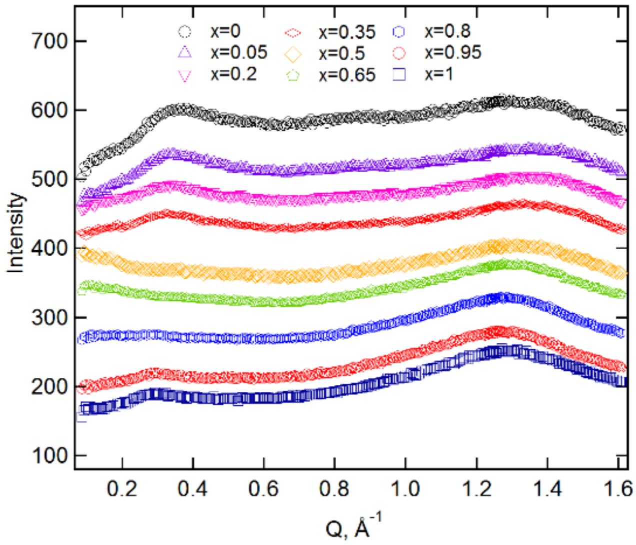

Figure S6: SANS data collected on D16 for $[h\text{-}C_8MIM]_{1-x}[C_8MIM\text{-}F_{13}]_x[Tf_2N]$ mixtures recorded at room temperature. Data have been offset in the y-axis to aid visualisation.

Fit parameters

Table S2: Fit parameters used to fit the SANS data for $[d_{17}\text{-}C_8MIM]_{1-x}[C_8MIM\text{-}F_{13}]_x[Tf_2N]$ using a combination of Lorentzian peaks and an Ornstein-Zernicke model to determine correlation length (CL)

x	PNPP Å^{-1}	HWHM Å^{-1}	COP Å^{-1}	HWHM Å^{-1}	CP Å^{-1}	HWHM, Å^{-1}	CL Å
**0.00**	0.34 ± 0.02	0.17 ± 0.01	0.85 ± 0.04	1.4 ± 0.08	1.38 ± 0.03	0.30 ± 0.02	-
**0.05**	0.33 ± 0.01	0.15 ± 0.01	0.85 ± 0.03	1.9 ± 0.1	1.36 ± 0.01	0.24 ± 0.02	-
**0.20**	0.32 ± 0.02	0.19 ± 0.01	0.86± 0.1	1.0± 0.10	1.34 ± 0.02	0.27± 0.02	-
**0.35**	0.33 ± 0.01	0.12 ± 0.01	-	-	1.33 ± 0.07	0.33± 0.02	1.1 ± 0.1
**0.50**	0.33 ± 0.02	0.11 ± 0.01	-	-	1.31 ± 0.03	0.31 ± 0.02	1.3 ± 0.2
**0.65**	0.32 ± 0.01	0.13± 0.01	-	-	1.30 ± 0.03	0.33 ± 0.03	1.1± 0.2
**0.80**	0.31 ± 0.02	0.15± 0.02	0.92 ± 0.20	0.79 ± 0.2	1.29 ± 0.01	0.27 ± 0.03	-
**0.95**	0.30 ± 0.02	0.13 ± 0.01	0.93 ± 0.04	2.89 ± 0.2	1.29 ± 0.07	0.30 ± 0.02	-
**1.00**	0.29 ± 0.02	0.11 ± 0.01	0.93 ± 0.09	3.3 ± 0.3	1.31 ± 0.02	0.33 ± 0.03	-

Table S3: Fit parameters used to fit the SANS data for for $[h\text{-}C_8MIM]_{1-x}[C_8MIM\text{-}F_{13}]_x[Tf_2N]$ using a combination of Lorentzian peaks and an Ornstein-Zernicke model to determine correlation length (CL)

x	PNPP Å^{-1}	HWHM Å^{-1}	COP Å^{-1}	HWHM Å^{-1}	CP Å^{-1}	HWHM, Å^{-1}	CL Å
**0.00**	0.34 ± 0.04	0.17 ± 0.03	0.85 ± 0.02	0.30 ± 0.02	1.36 ± 0.04	0.34 ± 0.04	-
**0.05**	0.33 ± 0.02	0.16 ± 0.02	0.79 ± 0.01	0.70 ± 0.03	1.40 ± 0.05	0.32 ± 0.05	
**0.20**	0.32 ± 0.02	0.24 ± 0.02	0.87 ± 0.02	0.36 ± 0.06	1.36 ± 0.02	0.30 ± 0.03	
**0.35**	0.33 ± 0.02	0.25± 0.02	0.88 ± 0.02	0.18 ± 0.06	1.34 ± 0.02	0.29 ± 0.01	
**0.50**	0.32 ± 0.02	0.5 ± 0.05	0.92 ± 0.06	0.30 ± 0.15	1.33 ± 0.03	0.37 ± 0.03	
**0.65**	-	-	-	-	1.30 ± 0.04	0.33 ± 0.03	1.8± 0.1
**0.80**	-	-	0.92± 0.10	0.13 ± 0.20	1.28 ± 0.03	0.28± 0.03	
**0.95**	0.30 ± 0.02	0.13 ± 0.02	0.93 ± 0.10	0.77 ±0.12	1.28 ± 0.02	0.30 ± 0.02	
**1.00**	0.29 ± 0.02	0.11 ± 0.01	0.93 ± 0.09	3.3 ± 0.3	1.31 ± 0.02	0.33 ± 0.03	-

SANS2d ISIS: Didcot UK

SANS was carried out on the Sans2d small-angle diffractometer at the ISIS Pulsed Neutron Source (STFC Rutherford Appleton Laboratory, Didcot, U.K.).[3,4] A collimation length of 4m and incident wavelength range of 1.75 – 16.5 Å was employed. Data were measured simultaneously on two 1 m^2 detectors to give a *q*-range of 0.0041 – 0.97 $Å^{-1}$. The small-angle detector was position 4m from the sample and offset vertically 60 mm and sideways 100 mm. The wide-angle detector was position 2.4m from the sample, offset sideways by 980 mm and rotated to face the sample. The scattering vector *q* is defined as:

$$q = \frac{4\pi \sin\frac{\theta}{2}}{\lambda}$$

where θ is the scattered angle and λ is the incident neutron wavelength. The beam diameter was 8 mm. Each raw scattering data set was corrected for the detector efficiencies, sample transmission and background scattering and converted to scattering cross-section data ($\partial\Sigma/\partial\Omega$ *vs q*) using the instrument-specific software.[5,6] These data were placed on an absolute scale (cm^{-1}) using the scattering from a standard sample (a solid blend of hydrogenous and perdeuterated polystyrene) in accordance with established procedures.[7] The data were fitted using SasView software.

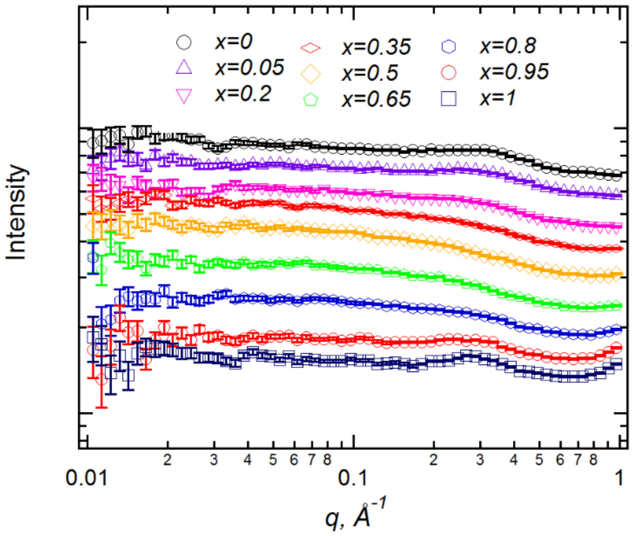

Figure S7: SANS data collected on SANS2D for [h-C_8MIM]$_{1-x}$[C_8MIM-F_{13}]$_x$[Tf_2N] mixtures recorded at room temperature. Data have been offset in the y-axis to aid visualisation.

Fit parameters

Table S4: Fit parameters used to fit the SANS data for $[d_{17}\text{-}C_8MIM]_{1-x}[C_8MIM\text{-}F_{13}]_x[Tf_2N]$ using a combination of Lorentzian peaks and an Ornstein-Zernicke model to determine correlation length (CL)

x	PNPP Å^{-1}	HWHM Å^{-1}	COP Å^{-1}	HWHM Å^{-1}	CP Å^{-1}	HWHM, Å^{-1}	CL Å
**0.00**	0.35 ± 0.01	0.16 ± 0.02			1.34 ± 0.10	0.3 ± 0.2	
**0.05**	0.33 ± 0.03	0.18 ± 0.01	-	-	1.35 ± 0.15	0.3 ± 0.1	-
**0.20**	0.31 ± 0.03	0.19± 0.02	-	-	1.36 ± 0.10	0.27 ± 0.1	-
**0.35**	0.33± 0.03	0.12 ± 0.02	-	-	1.34± 0.10	0.29± 0.06	1.75 ± 0.05
**0.50**	0.33 ± 0.04	0.15± 0.05	-	-	1.31± 0.09	0.31 ± 0.09	5.7 ± 1.5
**0.65**	0.32 ± 0.02	0.13 ± 0.03	-	-	1.30± 0.10	0.33 ± 0.05	5.1 ± 1.0
**0.80**	0.31 ± 0.03	0.19 ± 0.04	-	-	1.28± 0.04	0.28± 0.06	7.6 ± 1.6
**0.95**	0.30 ± 0.02	0.13 ± 0.02	-	-	1.28 ± 0.03	0.30 ± 0.01	7.3 ± 0.5
**1.00**	0.30 ± 0.01	0.12 ± 0.01	-	-	1.30 ± 0.10	0.34 ± 0.1	6.5 ± 0.8

Table S5: Fit parameters used to fit the SANS data for for $[h\text{-}C_8MIM]_{1-x}[C_8MIM\text{-}F_{13}]_x[Tf_2N]$ using a combination of Lorentzian peaks and an Ornstein-Zernicke model to determine correlation length (CL)

x	PNPP Å^{-1}	HWHM Å^{-1}	COP Å^{-1}	HWHM Å^{-1}	CP Å^{-1}	HWHM, Å^{-1}	CL Å
**0.00**	0.34 ± 0.03	0.20 ± 0.03	-	-	-	-	4.3 ± 1.0
**0.05**	0.33 ± 0.03	0.18 ± 0.03			1.39 ± 0.1	0.4 ± 0.2	4.1 ± 0.3
**0.20**	0.32 ± 0.01	0.19± 0.02	-	-	1.36 ± 0.1	0.27 ± 0.05	4.0 ± 0.8
**0.35**	-	-	0.88 ± 0.04	0.3 ± 0.05	1.34 ± 0.04	0.29 ± 0.04	1.5 ± 0.1
**0.50**	-	-	-	-	1.32 ± 0.15	0.32 ± 0.08	3.6 ± 0.3
**0.65**	-	-	-	-	1.30± 0.2	0.33 ± 0.15	3.4 ± 0.04
**0.80**	0.31 ± 0.2	0.19 ± 0.2	-	-	1.28 ± 0.08	0.28 ± 0.07	4.0 ±0.5
**0.95**	0.30 ± 0.02	0.12 ± 0.02	-	-	1.28 ± 0.05	0.30 ± 0.05	4.1 ± 0.8
**1.00**	0.29 ± 0.01	0.12 ± 0.01	-	-	1.30 ± 0.10	0.34 ± 0.1	6.5 ± 0.8

References

(1) Smoll, E. J.; Tesa-Serrate, M. A.; Purcell, S. M.; D'Andrea, L.; Bruce, D. W.; Slattery, J. M.; Costen, M. L.; Minton, T. K.; McKendrick, K. G. Determining the Composition of the Vacuum-Liquid Interface in Ionic-Liquid Mixtures. *Faraday Discuss.* **2018**, *206*, 497–522.

(2) Bara, J. E.; Gabriel, C. J.; Carlisle, T. K.; Camper, D. E.; Finotello, A.; Gin, D. L.; Noble, R. D. Gas Separations in Fluoroalkyl-Functionalized Room-Temperature Ionic Liquids Using Supported Liquid Membranes. *Chem. Eng. J.* **2009**, *147*, 43–50.

(3) ISIS Sans2d https://www.isis.stfc.ac.uk/Pages/Sans2d.aspx (accessed Apr 19, 2023).

(4) Heenan, R. K.; Rogers, S. E.; Turner, D.; Terry, A. E.; Treadgold, J.; King, S. M. Small Angle Neutron Scattering Using Sans2d. **2011**, *22*, 19–21.

(5) Arnold, O.; Bilheux, J. C.; Borreguero, J. M.; Buts, A.; Campbell, S. I.; Chapon, L.; Doucet, M.; Draper, N.; Ferraz Leal, R.; Gigg, M. A.; et al. Mantid—Data Analysis and Visualization Package for Neutron Scattering and μ SR Experiments. *Nucl. Instruments Methods Phys. Res. Sect. A Accel. Spectrometers, Detect. Assoc. Equip.* **2014**, *764*, 156–166.

(6) Mantid Project — MantidProject landing page documentation https://www.mantidproject.org/ (accessed Apr 19, 2023).

(7) Wignall, G. D.; Bates, F. S. Absolute Calibration of Small-Angle Neutron Scattering Data. *J. Appl. Crystallogr.* **1987**, *20*, 28–40.
